# Supplementary material for: Priority setting of vaccine introduction in Bangladesh: a multicriteria decision analysis study
Source: BMJ Open. 2022 Feb 28;12(2):e054219. doi: 10.1136/bmjopen-2021-054219 (PMC8886403; doi:10.1136/bmjopen-2021-054219)
Supplement: Supplementary data [file bmjopen-2021-054219supp001.pdf]

SUPPLEMENTARY

Supplementary A: Data collection instrument of Workshop A - Ranking of criteria

Name of the participants: ____

Designation: ____

Organization: ____

Note: Pls. make rank of the following criteria - 1 to 10 (Where is the most important and 10 is the less important)

CRITERIA	DEFINITION	RANK
**Criteria of Disease**		
Case fatality rate	Percentage of death among the cases	
Incidence rate of disease	Number of new cases per 100,000 population per year	
Outbreak potentiality	Potentiality of the disease to be epidemic Potentiality will be measured by the reproduction of the disease	
Severity of disease	Symptoms of the disease; how severe the disease are in the most of the cases	
Size of population at risk	Size of the population at risk or the target population for vaccination	
Type of Target population/ Demographic consideration	Demographic consideration or Target population for the vaccination against the disease (e.g. children or female or adult)	
**Criteria of Vaccine**		
Cost-effectiveness	Cost-effectiveness of vaccine; Incremental cost-effectiveness ration (ICER) will be $/QALY gained or $/DALY avoided if the vaccine introduced in comparison to the No vaccination	
Vaccine efficacy	Effectiveness of vaccine or the percentage reduction of diseases provided by vaccine	
**Other Criteria**		
Equity	Disease occur more in economically poor people or disadvantaged population	
Global Target	Global agenda of eradication/ elimination/ control target	

Supplementary B: Data collection instrument of Workshop C - Ranking of vaccines

Name of the participants: ____

Designation: ____

Organization: ____

Please rank vaccines from 1-7, where 1 is most favourable and 7 is least favourable

	Criteria	Rotavirus	HPV	Cholera	JE	Typhoid	Influenza	Dengue
DISEASE	**1. Incidence rate**<br>Number of new cases per 100,000 population per year	1080/100,000	24.3/100,000	210/100,000	2.7/100,000	280/100,000	10,000/ 100,000 person year (2008) 6600/100,000 person year (2009) 17000/100,000 person year (2010)	1340-5780/ 100,000 person-season
	**2. Case fatality rate**<br>Percentage of death among the cases	0.03% 12.42/100,000 among < 5 years of age (Rotavirus gastroenteritis mortality) (1.24%)	1.8%<br>50%	1.5%	10-30%<br>(25%)	0.3%<br>(1%)	0.08%	2.5%
VACCINE	Type of vaccine	RV5; Live attenuated	Human Papillomaviruses Nanovalent	Shanchol	SA14-14-2 JE Vaccine	Typhoid Conjugate vaccine	Influenza trivalent vaccine: Single dose	Dengvaxia live attenuated, recombinant tetravalent vaccine
	Dosage	3 dosages	2 dosages	2 dosages	Single dose	Single dose	Single dose	3 dosages
	**3. Vaccine efficacy**<br>Effectiveness of vaccine or the percentage reduction of diseases provided by vaccine	55%<br>(40-85%)	90-100%	50-60%	95%<br>>85%	50-72%	40%<br>40-60%	66%
POPULATION	**4. Type of Target population**<br>Demographic consideration or Target population for the vaccination against the disease (e.g. children or female or adult)	Under 5 population	Girls of 10 years of age or class 5 student<br>1.54 million	1-5years of population Urban and high risk population	1-15 years of children; Routine immunization 9-12 months	Under 5 population	High risk group	Dhaka City population
	**5. Size of population at risk (million)**<br>Number of population need to be vaccinated or size of the population at risk or the target population for vaccination	15.17	9.17<br>1.54	13.3	7.4<br>(1-15 years of children)	15.17	15.47	2.18
	RANK							
