# Supplementary material for: Priority setting of vaccine introduction in Bangladesh: a multicriteria decision analysis study
Source: BMJ Open. 2022 Feb 28;12(2):e054219. doi: 10.1136/bmjopen-2021-054219 (PMC8886403; doi:10.1136/bmjopen-2021-054219)
Supplement: Author's manuscript [file bmjopen-2021-054219.draft_revisions.pdf]

BMJ Open is committed to open peer review. As part of this commitment we make the peer review history of every article we publish publicly available.

When an article is published we post the peer reviewers' comments and the authors' responses online. We also post the versions of the paper that were used during peer review. These are the versions that the peer review comments apply to.

The versions of the paper that follow are the versions that were submitted during the peer review process. They are not the versions of record or the final published versions. They should not be cited or distributed as the published version of this manuscript.

BMJ Open is an open access journal and the full, final, typeset and author-corrected version of record of the manuscript is available on our site with no access controls, subscription charges or pay-per-view fees (http://bmjopen.bmj.com).

If you have any questions on BMJ Open's open peer review process please email info.bmjopen@bmj.com

BMJ Open

Priority Setting of Vaccines in Bangladesh using Multi-Criteria Decision Analysis

Journal:	*BMJ Open*
Manuscript ID	bmjopen-2021-054219
Article Type:	Original research
Date Submitted by the Author:	08-Jun-2021

Complete List of Authors:	Haider, Mohammad; Mahidol University, Health Technology Assessment Program; Government of Bangladesh Ministry of Health and Family Welfare, Directorate General of Health Services<br>Youngkong, Sitaporn; Mahidol University, Health Technology Assessment Program; Mahidol University, Social and Administrative Pharmacy Division, Department of Pharmacy, Faculty of Pharmacy<br>Thavorncharoensap, Montarat; Mahidol University, Health Technology Assessment Program; Mahidol University, Social and Administrative Pharmacy Division, Department of Pharmacy, Faculty of Pharmacy<br>Thokala, Praveen; The University of Sheffield, Health Economics and Decision Science; School of Health and Related Research (ScHARR)

Keywords:	HEALTH ECONOMICS, Health policy < HEALTH SERVICES ADMINISTRATION & MANAGEMENT, INFECTIOUS DISEASES, PUBLIC HEALTH

SCHOLARONE™
Manuscripts

I, the Submitting Author has the right to grant and does grant on behalf of all authors of the Work (as defined in the below author licence), an exclusive licence and/or a non-exclusive licence for contributions from authors who are: i) UK Crown employees; ii) where BMJ has agreed a CC-BY licence shall apply, and/or iii) in accordance with the terms applicable for US Federal Government officers or employees acting as part of their official duties; on a worldwide, perpetual, irrevocable, royalty-free basis to BMJ Publishing Group Ltd ("BMJ") its licensees and where the relevant Journal is co-owned by BMJ to the co-owners of the Journal, to publish the Work in this journal and any other BMJ products and to exploit all rights, as set out in our licence.

The Submitting Author accepts and understands that any supply made under these terms is made by BMJ to the Submitting Author unless you are acting as an employee on behalf of your employer or a postgraduate student of an affiliated institution which is paying any applicable article publishing charge ("APC") for Open Access articles. Where the Submitting Author wishes to make the Work available on an Open Access basis (and intends to pay the relevant APC), the terms of reuse of such Open Access shall be governed by a Creative Commons licence – details of these licences and which Creative Commons licence will apply to this Work are set out in our licence referred to above.

Other than as permitted in any relevant BMJ Author's Self Archiving Policies, I confirm this Work has not been accepted for publication elsewhere, is not being considered for publication elsewhere and does not duplicate material already published. I confirm all authors consent to publication of this Work and authorise the granting of this licence.

For peer review only

Manuscript Title: Priority Setting of Vaccines in Bangladesh using Multi-Criteria Decision Analysis

Sabbir Haider,

Mahidol University Health Technology Assessment (MUHTA) Graduate Program, Bangkok, Thailand
Directorate General of Health Services, Ministry of Health and Family Welfare, Dhaka, Bangladesh

Sitaporn Youngkong, PhD,

Mahidol University Health Technology Assessment (MUHTA) Graduate Program, Bangkok, Thailand
Social and Administrative Pharmacy Division, Department of Pharmacy, Faculty of Pharmacy, Mahidol University, Bangkok, Thailand

Montarat Thavorncharoensap, PhD,

Mahidol University Health Technology Assessment (MUHTA) Graduate Program, Bangkok, Thailand
Social and Administrative Pharmacy Division, Department of Pharmacy, Faculty of Pharmacy, Mahidol University,
Bangkok, Thailand

Praveen Thokala, PhD,

Health Economics and Decision Science,
School of Health and Related Research (ScHARR),
University of Sheffield,
Sheffield, England, UK

Contact information for corresponding author:

Sitaporn Youngkong,
Social and Administrative Pharmacy Division, Department of Pharmacy, Faculty of Pharmacy, Mahidol University,
447 SriAyudthaya Rd, Rachathewi, Bangkok 10400, Thailand
e-mail: sitaporn.you@mahidol.edu

Contributors: SH conceived and design the study, collected data, analyze the results and drafted the manuscript. SY and PT helped in design the study, analyze the results and reviewed the manuscript. MT contributed in design the study and reviewed the manuscript. All authors discussed the findings of the study, edited and approved the manuscript.

For peer review only

Financial disclosure: This research work was supported by funding from Mahidol University and the International Decision Support Initiative (iDSI) through the doctoral study in Mahidol University Health Technology Assessment (MUHTA) Graduate Program. This work was produced as part of the iDSI (www.idsihealth.org), which supports countries to get the best value for money from health spending. iDSI [grant number-OPP1087363] receives funding support from the Bill & Melinda Gates Foundation and the UK Department for International Development.

Disclaimer: The findings, interpretations and conclusions expressed in this article do not necessarily reflect the views of the funding agencies

Ethical consideration: Ethical clearance of this study was obtained from the Bangladesh Medical and Research Council (BMRC), Bangladesh (BMRC/NREC/206-2019/108 Date: 27-06-2019).

Data sharing: Data is available by emailing corresponding author Sitaporn Youngkong, email- sitaporn.you@mahidol.edu

Word count: Abstract: 240 ; Manuscript: 2875

Number Figures: 0

Number of Tables: 6

Appendix/Supplemental materials: 0

For peer review only

Manuscript Title: Priority Setting of Vaccines in Bangladesh using Multi Criteria Decision Analysis

ABSTRACT

INTRODUCTION

Decisions on new vaccine introduction in the health benefit package is challenging in resource limited countries such as Bangladesh. A number of criteria play a crucial role in the decision on which vaccines should be prioritised. The objective of the study was to prioritize vaccines for introduction in Bangladesh applying multi-criteria decision analysis (MCDA).

METHODS

MCDA process was applied to prioritize the potential vaccines for introduction in Bangladesh. A set of criteria was identified, weighted, and scores were assigned the different levels of the criteria. The performance matrix of the evaluation results was constructed against the criteria set. Relevant stakeholders participated in different steps based on the objective of each step. The vaccines were ranked and then appraised by stakeholders.

RESULTS

Five criteria including incidence rate, case fatality rate, vaccine efficacy, size of population at risk and type of population at risk were used quantitatively to evaluate and to score the vaccines. Upon deliberation, Japanese Encephalitis vaccine was the top ranked to be introduced in Bangladesh. MCDA supported to guide the national decision-makers with a scientific and evidence-based systematic process incorporating multiple criteria and involving related key stakeholders.

CONCLUSION

This study presented the first application of MCDA to support the vaccine prioritization in Bangladesh health system, and was based on systematic evidence-based decision-making.. Policy makers should promote the use of the method MCDA to prioritize interventions in healthcare, as the decision-making process can be improved using systematic MCDA approach.

Key Words: multi-criteria decision analysis, MCDA, priority setting, vaccine

For peer review only

For peer review only - http://bmjopen.bmj.com/site/about/guidelines.xhtml

ARTICLE SUMMARY

Strenghts and limitations of the study

- A number of criteria play a crucial role in the decision on which vaccines should be prioritised so that multi-criteria decision analysis (MCDA), a systematic and evidence-based approach, has been introduced in prioritising vaccines for introduction in Bangladesh.
- This vaccine prioritization in Bangladesh was participatory, transparent, accountable, and evidence-informed that ensured for a fair priority setting approach.
- Value judgment is still in need in decision made for vaccine prioritization.

For peer review only

For peer review only - http://bmjopen.bmj.com/site/about/guidelines.xhtml

MANUSCRIPT MAIN

INTRODUCTION

Vaccination is the most effective public health measure to prevent infectious diseases.[1 2] Governments in developing countries prefer to invest in vaccination programs which can be financially sustainable.[3-5] While countries often consider cost-effectiveness, this should not be the only criterion for the selection of any intervention.[6 7] Different criteria, such as disease severity, effectiveness, accessibility, quality of care and equity, should play significant role in priority setting in healthcare.[8]

Decision on new vaccines to be included in the benefit package is complex.[9] There are systematic and evidence-based methods,[10] using priority setting to allocate the scarce resources to meet increasing demands.[11] Multi-Criteria Decision Analysis (MCDA) is one such approach which evaluates different options considering multiple criteria in explicit manner,[12] to aid decision makers to take fair decisions.[13] MCDA can be a useful approach to support inclusion of health interventions in the benefit pacakage.[7]

Vaccine preventable diseases such as dengue, human papillomavirus (HPV), influenza, japanese encephalitis (JE), and typhoid, are prevalent in Bangladesh.[14-18] These diseases can be prevented by the introduction of new or underused vaccines by the government of Bangladesh. However, as new vaccines have considerable budget impact, it is not clear which of those should be prioritised. In the past, decision-making for vaccine introduction has been ad-hoc but there is increasing interest in prioritsation systematically evaluating multiple criteria.[19]

As such, we conducted a study applying MCDA to prioritise vaccines for introduction. This is the first study on prioritization of health interventions to make better use limited resources in Bangladesh, which provided the national decision makers of ministry of health with a scientific and evidence-based systematic process incorporating multiple criteria and involving related key stakeholders. This paper describes the methods and results of the study, along with discussion and conclusions.

METHODS

We followed the steps outlined in good practice guidelines for the use of MCDA in health care.[20 21] As stakeholder involvement is key, we conducted four workshops (between October 2019 and January 2020) with the relevant stakeholders during the MCDA process. The steps and the workshops are described in further detail below.

For peer review only - http://bmjopen.bmj.com/site/about/guidelines.xhtml

1. Identifying the list of potential vaccines for introduction

The potential vaccines for prioritization were identified from the recommendations of World Health Organization (WHO), Gavi the vaccine alliance, and centers for disease control and prevention in the USA (CDC-US). The vaccines which were currently in the expanded program on immunization (EPI) program of the neighbouring countries were also collected. From these sources, vaccines which were not yet introduced in Bangladesh were identified to select the list of potential vaccines to be evaluated.

2. Selecting criteria for vaccine introduction in Bangladesh

A three step process was used to select criteria for vaccine introduction in Bangladesh. First, a systematic review was conducted to identify all potential criteria for vaccine introduction in Bangladesh, which is described elsewhere in detail.[22] Second, from this long list of criteria, core team of three public health experts of Bangladesh (including the lead author, SH) excluded criteria that were not relevant to put together a list of potential criteria. Criteria that cannot be quantified (e.g. political will) and the criteria that were mentioned less frequently were excluded.

Finally, the potential criteria list were ranked in a workshop A (WS-A) in October 2019, to identify the key criteria to be used for prioritisation of vaccines. Stakeholders included paediatricians, public health experts, virologists, epidemiologists, health economics and health system experts from directorate offices, technical institutes, non-government ofrganizations (NGOs), national immunization technical advisory group (NITAG), and health professional associations. The criteria, along with their definitions, were presented to the stakeholders in the WS-A. Stakeholders were then asked to rank the criteria from '1 to 10', where '1' is the most preferable and '10' is the least preferable criterion. The ranked order of criteria was transformed into mean ranks using rank order centroid (ROC) method.[23] Based on the mean rankings, stakeholders selected a set of criteria by consensus to be used in prioritising vaccines.

3. Weighting and scoring

In the same workshop (WS-A), the stakeholders weighted the criteria using direct rating methods. Stakeholders discussed and then agreed by consensus to assign points to each criterion in a scale of 0 -100, where '0' depicted the least important, and '100' represented the most important. To calculate the weights, the points assigned for each criterion was normalized

For peer review only - http://bmjopen.bmj.com/site/about/guidelines.xhtml

(i.e. by dividing the points allocated to each criterion by the sum of points of all criteria) using Equation 1.[24 25]

$$w_i = p_i / \sum p_i \qquad \text{Equation 1}$$

where, w_i = normalized weight of criterion i

i = index of criterion

p_i = points allocated to each criterion

For scoring, the levels of criteria were identified by the core team from literature review and expert opinion. These were presented to the stakeholders in WS-A, who then assigned scores to the levels in each criteria individually. The stakeholders deliberated on these individual scores and assigned scores to each level of the criteria by consensus. The range of scores were between 0 to 1, where, '0' depicted the lowest score, and '1' represented the highest score.

4. Gathering evidence

Data for the potential vaccines were collected from literature reviews, databases and reports from key organisations such as EPI, Communicable Disease Control of Directorate General of Health Services (CDC-DGHS), Institute of Epidemiology, Disease Control and Research (IEDCR), and International Centre for Diarrhoeal Disease and Research, Bangladesh (icddr,b). A performance matrix was constructed, which presents data of each vaccine against the set of criteria. Then another workshop B (WS-B) was arranged in November 2019, to validate the data with a group of public health and vaccine experts in the country, i.e., public health experts who were working in the national vaccination programs, and disease surveillance from CDC-DGHS, health economics unit (HEU) and NITAG. After reviewing and validation, they signed off on the performance matrix.

5. Rank ordering the potential vaccines

The scores for the different levels from the WS-A were combined with the validated performance matrix from the WS-B to calculate the scores for each vaccine on the different criteria. Then, using the additive method[21] (see Equation 2),[26] the scores of each vaccine corresponding to the criteria level was multiplied by the weight of each criterion to

For peer review only

For peer review only - http://bmjopen.bmj.com/site/about/guidelines.xhtml

calculate the total scores of each potential vaccine. The vaccines were ranked based on the total scores of each vaccine, with the highest total score ranked top, and the next highest total second, and so on.

$$V_j = \sum C_{ij} * W_i \qquad \text{Equation 2}$$

Where V_j is the total value for alternative i,

C_{ij} is the score of level on criteria i and

W_i is the weight attached to criteria i.

6. Appraising the rank of vaccines

Another workshop C (WS-C) was arranged in December 2019 with the experts in the area of vaccination, i.e., epidemiologists, virologists, public health specialists, surveillance experts, and members of the vaccination policy program. The performance matrix of potential vaccines was provided in paper-based format and the stakeholders were asked to assign the rank to the seven potential vaccines individually, where '1' is the most preferable vaccine. The mean rank of each vaccine were calculated from the ranks provided by each stakeholder, using the ROC method (equation 1).

The ranking analysis of vaccines retrieved from step 5 based on findings from WS-A and WS-B were then presented to the stakeholders, along with the evidence of the cost-effectiveness and outbreak potentiality of each vaccine. Stakeholders then considered all this information and deliberated to reach consensus on a final ranking of vaccines.

7. Application of vaccine prioritization process in Bangladesh health system

A final workshop D (WS-D) was organised in January 2020 with the policy makers working at the ministry of health in vaccine decision-making, vaccination program implementation, vaccine related research, and disease surveillance. This workshop involved dissemination of the whole vaccine prioritization process (including the selection process of criteria, identification of vaccines and the MCDA methods), along with the findings. The list of ranked ordered vaccines was submitted to the ministry of health of Bangladesh for further policy action.

For peer review only - http://bmjopen.bmj.com/site/about/guidelines.xhtml

Patient and public involvement

In this study, patients were not involved or participated.

RESULTS

1. The list of potential vaccines for introduction in Bangladesh

WHO recommended 23 vaccines for introduction as routine vaccination globally, whereas the CDC-US recommended 16 vaccines and Gavi the vaccine alliance provided support against 16 infectious diseases.[27-29] Bangladesh so far introduced 10 vaccines in their benefit package and two additional vaccines for the Haj pilgrimage travellers. Therefore, there were 11 vaccines not included yet in the Bangladesh health benefit package. After discussion among the core team and vaccine experts, vaccines were excluded for four conditions (tick-borne encephalitis, and yellow fever as Bangladesh lacked incidence data for these diseases, and varicella and hepatitis-A virus vaccines as they were not included in the benefit package of the neighbouring countries). Seven vaccines (i.e., cholera, dengue, typhoid, HPV, influenza, JE, and rotavirus) were selected for consideration in the priority setting process.

2. Prioritization criteria for vaccine introduction in Bangladesh

Sixty-seven criteria were identified in the systematic review, from which the core team identified 10 criteria as being potentially most relevant (Table 1). Definitions of these 10 criteria were collected from the WHO[30 31] and Bangladesh.[32]

In the workshop WS-A, stakeholders discussed the importance of each of these 10 criteria and justification for its inclusion in the set of prioritization criteria to be used for vaccine introduction in Bangladesh. Participants ranked individually first and after deliberation, consensus was achieved. Table 1 presents the mean of individual ranking using ROC method and the final consensus ranking. Based on these rankings, stakeholders selected the top five criteria for vaccine prioritisation in Bangladesh (i.e., incidence rate, case fatality rate, vaccine efficacy, size of population at risk, and type of population at risk). In addition to these five quantitative criteria, stakeholders also decided to discuss qualitatively 'outbreak potentiality' and 'cost-effectiveness' criteria. These two criteria were not weighted or scored explicitly, but the vaccines performance against these criteria were used in deliberative discussions.

For peer review only - http://bmjopen.bmj.com/site/about/guidelines.xhtml

3. Performance matrix

The data collected on the performance of each of seven vaccines against the prioritization criteria were presented in Table 2. The table presents the data on the five quantitative criteria used for weighting and scoring, as well as the two qualitative criteria that were used in deliberative discussions.

4. Weighting and scoring

The participants of the WS-A consensually assigned 100 points to the criterion of incidence rate and four other criteria were assigned points in accordance, with the least important criterion, 'type of population at risk' assigned 50 points. The weight of each criterion was calculated by using the linear normalization method, where weights of 'incidence rate' and 'size of population at risk' were 0.26 and 0.19, respectively as presented in Table 3.

In the same workshop WS-A, the stakeholders assigned scores for the levels of each of the five criteria by consensus, using direct rating methods. The scores for the different levels of each criterion are presented in Table 4.

5. Rank ordering the potential vaccines

After combining the findings from Tables 2-4 (i.e. the weights and scores for the different levels from WS-A, and the performance matrix validated in WS-C), the core team performed analysis of seven vaccines and produced the ranking results, where cholera vaccine was top-ranked with the highest total score of 0.34 as shown in Table 5.

6. Appraising the rank of vaccines

In the WS-C, the stakeholders reviewed the performance matrix and each stakeholder ranked the vaccines individually first. The mean of their individual rankings are presented in Table 6.

The results of ranking by the core team (Table 5 using findings from WS-A and WS-B) were presented along with the information on potentiality of outbreak of the diseases and cost-effectiveness (see Table 2). After considering all this information, the stakeholders adjusted the ranking by consensus and the final ranking is presented in Table 6.

For peer review only - http://bmjopen.bmj.com/site/about/guidelines.xhtml

7. Application of vaccine prioritization process in Bangladesh health system

Twenty-eight stakeholders participated in the final dissemination workshop (WS-D), including representatives from the ministry of health, directorate office of health, development partners, health professional associations, primary health care, and NGOs. The stakeholders participated in discussions on both prioritization process and the ranking of vaccines. Decision-makers outlined importance on the appraising new interventions scientifically and agreed to apply MCDA in the priority setting process in vaccine introduction decision making. They agreed on the importance of introduction on the JE vaccine as the top ranked vaccine in the government benefit package. The key personnel of ministry of health and family welfare, Bangladesh, stated –*"It is better for Bangladesh at present to have this system to prioritize vaccines in the country. Bangladesh, a low-middle income country is graduating Gavi funding. So, we have to change our decision-making process from donor influenced decision-making to self-decision-making."*. They also highlighted that after selection of vaccines, country should prepare for vaccine logistics such as cold-chain capacity and other programmatic issues.

DISCUSSION

This study represents the first time an explicit priority setting process based on MCDA was used to select the vaccines in Bangladesh. Vaccines selected for prioritisation were those which were recommended by the international organizations but not included in health benefit package of Bangladesh. Long list of multiple criteria were identified systematically from published literature, which were then shortlisted in two phases to select five quantitative criteria and two qualitative criteria for the evaluation of the vaccines. Weighting and scoring of the quantitative criteria were explicit and participatory, and the tool used for eliciting scores and weights were user friendly and well understood by the stakeholders. The final ranking of the vaccines was determined after considering the performance matrix, the ranking using quantitative criteria and the information on the qualitative criteria. The stakeholders decided unanimously to introduce JE vaccine in the government package (please note that the ranking of vaccines and the selection of JE vaccine is country specific and may not be applicable to other settings).

The MCDA process was supported by different stakeholders who are involved in the decision-making process of the country. Members of the different decision-making committees (NITAG), implementing bodies (EPI and others), and health professional

For peer review only - http://bmjopen.bmj.com/site/about/guidelines.xhtml

associations were involved in every step of the decision-making in this research. Stakeholders of implementing agencies – EPI and CDC-DGHS – also participated in the deliberative process and ranking. NITAG members and members of NCIP also participated in the final decision-making workshop at ministry level. Participation of stakeholders in this research ensured the transparency and accountability of decision-making, which is essential for a fair priority setting approach.[33]

Incidence rate of the disease and case fatality rate criteria weighted highly, indicating that disease burden was considered important for vaccine selection by the stakeholders. This finding is similar to other studies which suggest disease burden as the most common and important criterion considered by other low- and middle-income countries during national decision-making.[34 19 35-38] Efficacy of the vaccines was the next most important criterion suggesting that clinical effectiveness is also important. However, it should be noted that the final ranking was based on deliberation where the weights and scores were not explicit.

The stakeholders in the WS-C ranked the vaccines after a deliberative process reviewing the performance matrix, and their ranking was different from the ranking from quantitative weighting and scoring (from WS-A and WS-B). This may be due to the differences in the stakeholder membership between the different workshops and the underlying differences in their preferences. Also, this may be due to the preferences being implicit in the WS-C while they were explicitly elicited in the previous ranking. This highlights the importance of ensuring a consistent group of stakeholders and a consistent preference elicitation methodology throughout the MCDA process. If the membership or the methodology changes between the different workshops, there is a possibility that the ranking may change quite substantially.

Also, the ranking was finalised after considering the cost-effectiveness and the outbreak potentiality criteria, as well as the quantitative ranking. This was only slightly different to the ranking just from deliberative discussions of performance matrix, suggesting that the stakeholders were not influenced by the ranking from quantitative weighting and scoring, but rather from reviewing the cost-effectiveness results and data of outbreak potentiality. It is important to note that cost-effectiveness is not recommended as a criterion in the MCDA,[39 40] as such, a pragmatic approach was taken to consider this information qualitatively rather than weighting and scoring.

The final ranking in this study was based on the performance matrix. This construction of the performance matrix from the scientific analysis is one of the important steps, and observing data of all vaccines against the selected criteria is critical for informed appraisal. Deliberation among stakeholders followed by simple ranking appears a feasible

For peer review only - http://bmjopen.bmj.com/site/about/guidelines.xhtml

strategy for the prioritsation of vaccines for introduction in Bangladesh. This is also inline with the recent consensus on the use of MCDA for HTA,[41] which recommends deliberative MCDA approach over quantitative MCDA.

CONCLUSION

This study presents the first application of MCDA to support the vaccine prioritization in Bangladesh health system, and was based on systematic evidence-based decision-making. This research involved relevant stakeholders in priority setting process, and achieved the objectives for prioritizing the vaccine for introduction in Bangladesh in a transparent way. Policy makers should promote the use of the method MCDA to prioritize interventions in healthcare, as the decision-making process can be improved using systematic MCDA approach.

For peer review only - http://bmjopen.bmj.com/site/about/guidelines.xhtml

References

1. Timmis JK, Black S, Rappuoli R. Improving accountability in vaccine decision-making. *Expert Rev Vaccines* 2017;16(11):1057-66. doi: 10.1080/14760584.2017.1382358

2. Brenzel L, Wolfson LJ, Fox-Rushby J, et al. Vaccine-Preventable Diseases—Chapter 20. *Disease Control Priorities in Developing Countries*:389-411.

3. Ehreth J. The value of vaccination: a global perspective. *Vaccine* 2003;21(27-30):4105-17.

4. Ehreth J. The global value of vaccination. *Vaccine* 2003;21(7-8):596-600.

5. Mauskopf J, Standaert B, Connolly MP, et al. Economic Analysis of Vaccination Programs: An ISPOR Good Practices for Outcomes Research Task Force Report. *Value Health* 2018;21(10):1133-49. doi: 10.1016/j.jval.2018.08.005

6. Baltussen R, Jansen MP, Mikkelsen E, et al. Priority Setting for Universal Health Coverage: We Need Evidence-Informed Deliberative Processes, Not Just More Evidence on Cost-Effectiveness. *Int J Health Policy Manag* 2016;5(11):615-18. doi: 10.15171/ijhpm.2016.83

7. Norheim OF. Ethical priority setting for universal health coverage: challenges in deciding upon fair distribution of health services. *BMC Med* 2016;14:75. doi: 10.1186/s12916-016-0624-4

8. Youngkong S, Kapiriri L, Baltussen R. Setting priorities for health interventions in developing countries: a review of empirical studies. *Trop Med Int Health* 2009;14(8):930-9. doi: 10.1111/j.1365-3156.2009.02311.x

9. Munira SL, Fritzen SA. What influences government adoption of vaccines in developing countries? A policy process analysis. *Soc Sci Med* 2007;65(8):1751-64. doi: 10.1016/j.socscimed.2007.05.054

10. Baltussen R, Niessen L. Priority setting of health interventions: the need for multi-criteria decision analysis. *Cost Eff Resour Alloc* 2006;4:14. doi: 10.1186/1478-7547-4-14

For peer review only - http://bmjopen.bmj.com/site/about/guidelines.xhtml

11. Mitton C, Donaldson C. Health care priority setting: principles, practice and challenges. *Cost Eff Resour Alloc* 2004;2(1):3. doi: 10.1186/1478-7547-2-3

12. Thokala P, Duenas A. Multiple criteria decision analysis for health technology assessment. *Value Health* 2012;15(8):1172-81. doi: 10.1016/j.jval.2012.06.015

13. Marsh K, Goetghebeur M, Thokala P, et al. Multi-Criteria Decision Analysis to Support Healthcare Decisions: Springer 2017.

14. Haque F, Hossain MJ, Kundu SK, et al. Cholera Outbreaks in Urban Bangladesh In 2011. *Epidemiology (Sunnyvale)* 2013;3 doi: 10.4172/2161-1165.1000126

15. Hossain MJ, Gurley ES, Montgomery S, et al. Hospital-based surveillance for Japanese encephalitis at four sites in Bangladesh, 2003-2005. *Am J Trop Med Hyg* 2010;82(2):344-9. doi: 10.4269/ajtmh.2010.09-0125

16. Paul RC, Rahman M, Gurley ES, et al. A novel low-cost approach to estimate the incidence of Japanese encephalitis in the catchment area of three hospitals in Bangladesh. *Am J Trop Med Hyg* 2011;85(2):379-85. doi: 10.4269/ajtmh.2011.10-0706

17. Hasan AZ, Saha S, Saha SK, et al. Using pneumococcal and rotavirus surveillance in vaccine decision-making: A series of case studies in Bangladesh, Armenia and the Gambia. *Vaccine* 2018;36(32 Pt B):4939-43. doi: 10.1016/j.vaccine.2018.06.001

18. Chowdhury PDP, K..K.; Haque, C.E.; Hossain, S.; Lindsay, L. R.; Dibernardo, A.; Brooks, W. A.; Drebot, M. A. Dengue seroprevalence, seroconversion and risk factors in Dhaka, Bangladesh. *PLoS Negl Trop Dis* 2017;11(3) doi: https://doi.org/10.1371/journal.

19. Uddin J, Sarma H, Bari TI, et al. Introduction of new vaccines: decision-making process in Bangladesh. *J Health Popul Nutr* 2013;31(2):211-7.

For peer review only - http://bmjopen.bmj.com/site/about/guidelines.xhtml

20. Marsh K, M IJ, Thokala P, et al. Multiple Criteria Decision Analysis for Health Care Decision Making--Emerging Good Practices: Report 2 of the ISPOR MCDA Emerging Good Practices Task Force. *Value Health* 2016;19(2):125-37. doi: 10.1016/j.jval.2015.12.016

21. Thokala P, Devlin N, Marsh K, et al. Multiple Criteria Decision Analysis for Health Care Decision Making--An Introduction: Report 1 of the ISPOR MCDA Emerging Good Practices Task Force. *Value Health* 2016;19(1):1-13. doi: 10.1016/j.jval.2015.12.003

22. Haider MS. Multi-Criteria Decision Analysis for Priority Setting of Vaccine Introduction in Bangladesh [PhD Thesis]. Mahidol University, 2020.

23. Roszkowska E. Rank ordering criteria weighting methods–a comparative overview. 2013

24. Podviezko A, Podvezko V. Influence of data transformation on multicriteria evaluation result. *Procedia Engineering* 2015;122:151-57.

25. Normalization techniques for multi-criteria decision making: analytical hierarchy process case study. doctoral conference on computing, electrical and industrial systems; 2016. Springer.

26. Marsh K, Thokala P, Mühlbacher A, et al. Incorporating Preferences and Priorities into MCDA: Selecting an Appropriate Scoring and Weighting Technique. In: Marsh K, Goetghebeur M, Thokala P, et al., eds. Multi-Criteria Decision Analysis to Support Healthcare Decisions. Cham: Springer International Publishing 2017:47-66.

27. World Health Organization. WHO recommendations for routine immunization - summary tables 2019 [updated 26 April 2019; cited 2019 21 November 2019]. Available from: https://www.who.int/immunization/policy/immunization_tables/en/ accessed 21 November, 2019 2019.

For peer review only - http://bmjopen.bmj.com/site/about/guidelines.xhtml

28. Gavi The Vaccine Alliance. Gavi, The Vaccine Alliance-Vaccine Support 2019 [cited 2019 21 November 2019]. Available from: https://www.gavi.org/programmes-impact/types-support/vaccine-support2019.

29. Centers for Disease Control and Prevention. Centers for Disease Control and Prevention 2019 [Available from: https://www.cdc.gov/vaccines/ accessed 21 November 2019 2019.

30. World Health Organization. Global reference list of 100 core health indicators: World Health Organization, 2015.

31. World Health Organization. World Health Statistics 2012 - Indicator compendium: World Health Organization 2012.

32. Rashid K. Hyder's Textbook of community Medicine and public Health: RHM publishers, Dhaka, 2008.

33. Daniels N. Accountability for reasonableness. *BMJ* 2000;321(7272):1300-1.

34. Burchett HE, Mounier-Jack S, Griffiths UK, et al. National decision-making on adopting new vaccines: a systematic review. *Health Policy Plan* 2012;27 Suppl 2:ii62-76. doi: 10.1093/heapol/czr049

35. Hadisoemarto PF, Reich MR, Castro MC. Introduction of pentavalent vaccine in Indonesia: a policy analysis. *Health Policy Plan* 2016;31(8):1079-88. doi: 10.1093/heapol/czw038 [published Online First: 2016/04/24]

36. Kant L. NTAGI subcommittee recommendations on Haemophilus influenzae type b (Hib) vaccine introduction in India. *Indian Pediatr* 2009;46(11):945-54.

37. Makinen M, Kaddar M, Molldrem V, et al. New vaccine adoption in lower-middle-income countries. *Health Policy Plann* 2012;27(SUPPL.2):ii39-ii49. doi: 10.1093/heapol/czs036

For peer review only - http://bmjopen.bmj.com/site/about/guidelines.xhtml

38. Van Der Putten IM, Evers SMAA, Deogaonkar R, et al. Stakeholders' perception on including broader economic impact of vaccines in economic evaluations in low and middle income countries: A mixed methods study. *BMC Public Health* 2015;15(1) doi: 10.1186/s12889-015-1638-0

39. Marsh KD, Sculpher M, Caro JJ, et al. The Use of MCDA in HTA: Great Potential, but More Effort Needed. *Value in Health* 2018;21(4):394-97. doi: https://doi.org/10.1016/j.jval.2017.10.001

40. Marsh K, Thokala P, Youngkong S, et al. Incorporating MCDA into HTA: challenges and potential solutions, with a focus on lower income settings. *Cost Effectiveness and Resource Allocation* 2018;16(1):43. doi: 10.1186/s12962-018-0125-8

41. Baltussen R, Marsh K, Thokala P, et al. Multicriteria Decision Analysis to Support Health Technology Assessment Agencies: Benefits, Limitations, and the Way Forward. *Value in Health* 2019;22(11):1283-88. doi: https://doi.org/10.1016/j.jval.2019.06.014

42. Khan AI, Levin A, Chao DL, et al. The impact and cost-effectiveness of controlling cholera through the use of oral cholera vaccines in urban Bangladesh: A disease modeling and economic analysis. *PLoS Negl Trop Dis* 2018;12(10):e0006652. doi: 10.1371/journal.pntd.0006652

43. World Health Organization. Cholera 2014. *Weekly Epidemiological Record* 2015;90(40):517-44.

44. Hossain MA, Latif AHMM, Rayhan MI, et al. Population Projection of Bangladesh: Dynamics and Trends 2011-2061. Dhaka, Bangladesh: Bangladesh Bureay of Statistics, Statistics and Information Division, Ministry of Planning 2015.

45. Eisenberg MC, Robertson SL, Tien JH. Identifiability and estimation of multiple transmission pathways in cholera and waterborne disease. *J Theor Biol* 2013;324:84-102. doi: 10.1016/j.jtbi.2012.12.021

For peer review only - http://bmjopen.bmj.com/site/about/guidelines.xhtml

For peer review only

46. Cost-effectiveness of Dengue vaccine Introduction in Dhaka City, Bangladesh. HTAsiaLink 2018; 2018; Chiangmai, Thialand.

47. (WHO) WHO. Dengue vaccine: WHO position paper – July 2016, 2016:349-64.

48. Estimation of parameters and basic reproductive ratio for japanese encephalitis transmission in the philippines using a sequential monte carlo filter. 2017 IEEE Conference on Control Technology and Applications (CCTA); 2017. IEEE.

49. Mahumud RA, Gow J, Alam K, et al. Cost-effectiveness of the introduction of two-dose bi-valent (Cervarix) and quadrivalent (Gardasil) HPV vaccination for adolescent girls in Bangladesh. *Vaccine* 2020;38(2):165-72. doi: 10.1016/j.vaccine.2019.10.037

50. Services DGoH. National Strategy for Cervical Cancer Prevention and Control Bangladesh (2017-2022): Directorate General of Health Services, 2017.

51. Jit M, Brisson M, Portnoy A, et al. Cost-effectiveness of female human papillomavirus vaccination in 179 countries: a PRIME modelling study. *Lancet Glob Health* 2014;2(7):e406-14. doi: 10.1016/S2214-109X(14)70237-2

52. Riesen M, Garcia V, Low N, et al. Modeling the consequences of regional heterogeneity in human papillomavirus (HPV) vaccination uptake on transmission in Switzerland. *Vaccine* 2017;35(52):7312-21. doi: 10.1016/j.vaccine.2017.10.103

53. Ahmed M, Aleem MA, Roguski K, et al. Estimates of seasonal influenza-associated mortality in Bangladesh, 2010-2012. *Influenza Other Respir Viruses* 2018;12(1):65-71. doi: 10.1111/irv.12490

54. Brooks WA, Goswami D, Rahman M, et al. Influenza is a major contributor to childhood pneumonia in a tropical developing country. *Pediatr Infect Dis J* 2010;29(3):216-21. doi: 10.1097/INF.0b013e3181bc23fd

For peer review only

55. Zaman K, Roy E, Arifeen SE, et al. Effectiveness of maternal influenza immunization in mothers and infants. *The New England journal of medicine* 2008;359(15):1555-64. doi: 10.1056/NEJMoa0708630 [published Online First: 2008/09/19]

56. Orenstein EW, Orenstein LA, Diarra K, et al. Cost-effectiveness of maternal influenza immunization in Bamako, Mali: A decision analysis. *PLoS One* 2017;12(2):e0171499. doi: 10.1371/journal.pone.0171499

57. van den Driessche P. Reproduction numbers of infectious disease models. *Infect Dis Model* 2017;2(3):288-303. doi: 10.1016/j.idm.2017.06.002

58. Touch S, Suraratdecha C, Samnang C, et al. A cost-effectiveness analysis of Japanese encephalitis vaccine in Cambodia. *Vaccine* 2010;28(29):4593-9. doi: 10.1016/j.vaccine.2010.04.086

59. Organization WH. Japanese encephalitis vaccines: WHO position paper. *Weekly Epidemiological Record= Relevé épidémiologique hebdomadaire* 2015;90(09):69-88.

60. Tandan JB, Ohrr H, Sohn YM, et al. Single dose of SA 14-14-2 vaccine provides long-term protection against Japanese encephalitis: a case-control study in Nepalese children 5 years after immunization. drjbtandan@yahoo.com. *Vaccine* 2007;25(27):5041-5. doi: 10.1016/j.vaccine.2007.04.052

61. Sarker AR, Sultana M, Mahumud RA, et al. Cost-effectiveness analysis of introducing universal childhood rotavirus vaccination in Bangladesh. *Hum Vaccin Immunother* 2018;14(1):189-98. doi: 10.1080/21645515.2017.1356962

62. Troeger C, Khalil IA, Rao PC, et al. Rotavirus Vaccination and the Global Burden of Rotavirus Diarrhea Among Children Younger Than 5 Years. *JAMA Pediatr* 2018;172(10):958-65. doi: 10.1001/jamapediatrics.2018.1960

63. Zaman K, Dang DA, Victor JC, et al. Efficacy of pentavalent rotavirus vaccine against severe rotavirus gastroenteritis in infants in developing countries in Asia: a

For peer review only

For peer review only - http://bmjopen.bmj.com/site/about/guidelines.xhtml

randomised, double-blind, placebo-controlled trial. *Lancet* 2010;376(9741):615-23. doi: 10.1016/S0140-6736(10)60755-6

64. Antillon M, Bilcke J, Paltiel AD, et al. Cost-effectiveness analysis of typhoid conjugate vaccines in five endemic low- and middle-income settings. *Vaccine* 2017;35(27):3506-14. doi: 10.1016/j.vaccine.2017.05.001

65. Marchello CS, Hong CY, Crump JA. Global typhoid fever incidence: A systematic review and meta-analysis. *Clinical Infectious Diseases* 2019;68:S105-S16. doi: 10.1093/cid/ciy1094

66. Yu AT, Amin N, Rahman MW, et al. Case-Fatality Ratio of Blood Culture-Confirmed Typhoid Fever in Dhaka, Bangladesh. *J Infect Dis* 2018;218(suppl_4):S222-S26. doi: 10.1093/infdis/jiy543

67. Shakya M, Colin-Jones R, Theiss-Nyland K, et al. Phase 3 Efficacy Analysis of a Typhoid Conjugate Vaccine Trial in Nepal. *The New England journal of medicine* 2019;381(23):2209-18. doi: 10.1056/NEJMoa1905047

68. Pitzer VE, Bowles CC, Baker S, et al. Predicting the impact of vaccination on the transmission dynamics of typhoid in South Asia: a mathematical modeling study. *PLoS Negl Trop Dis* 2014;8(1):e2642. doi: 10.1371/journal.pntd.0002642

For peer review only

For peer review only - http://bmjopen.bmj.com/site/about/guidelines.xhtml

Table 1: Selecting criteria based on ranking from the workshop WS-A

Criteria	Rank	
	Using mean of individuals	Consensus after deliberation
Incidence rate of disease*	1	1
Case fatality rate*	2	2
Vaccine efficacy*	3	3
Size of population at risk*	5	4
Type of Target population/ Demographic consideration*	6	5
Outbreak potentiality	4	6
Cost-effectiveness	7	7
Severity of disease	8	8
Global Target	9	9
Equity	10	10

*criteria selected for vaccine prioritisation in Bangladesh

For peer review only

For peer review only - http://bmjopen.bmj.com/site/about/guidelines.xhtml

Table 2: Performance matrix with data of vaccines on the criteria validated in WS-B

Vaccine preventable disease	Incidence rate	Case fatality rate	Vaccine efficacy	Population at risk	Target population	Cost effectiveness**	Outbreak Potential***
	Number of new cases per 100,000 population per year	Percentage of death among the cases in a year	Effectiveness of vaccine or reduction of diseases provided by vaccine (%)	No. of population at risk of getting infection per year (in millions)	Type of population needed to be vaccinated	Cost-effectiveness results from published literature	
**Cholera**[42-45]	1640	3.0%	53	15.175	Under 5 children	cost-effective	High
**Dengue**[46-48]	3700	0.16% *	66	2.18 *	Adult/High-risk	Very cost-effective	High
**HPV**[49-52]	10.6	0.0115%	95	1.56	Woman	Highly cost-effective	Low
**Influenza**[44 53-57]	10,200	0.088%	63	15.5	High risk	cost-effective	Low
**Japanese encephalitis**[16 44 48 58-60]	2.7	30.0%	96.20	10.77	High risk	very cost effective	Medium
**Rotavirus**[44 61-63]	1080	0.0055%	43	15.175	Under 5 children	Very cost effective	High*
**Typhoid**[44 64-68]	280	0.30%	81.60	15.175	Under 5 children	Cost effective	Medium

* Expert opinion; **Not included in weighting and scoring, used in deliberative discussions in workshop WS-C for final rankings. Judgements on cost-effectiveness were made from conclusions from published literature which evaluated the cost-effectivenss of these vaccines in Bangladesh or similar countries. ***Not included in weighting and scoring, used in deliberative discussions in workshop WS-C for final rankings.

For peer review only - http://bmjopen.bmj.com/site/about/guidelines.xhtml

Table 3: Points allocated, and the calculated weights, for the criteria (from WS-A)

Criteria	Points	Weight
Incidence rate	100	0.26
Case fatality rate	85	0.22
Vaccine efficacy	80	0.21
Size of population at risk	75	0.19
Type of population at risk	50	0.13

For peer review only

Table 4: Scores for the levels of criteria (from WS-A)

Criteria	Levels	Score
**Incidence rate**	Level 1: >1000/100,000	1.0
	Level 2: 100-1000/100,000	0.8
	Level 3: 10-100/100,000	0.5
	Level 4: <10/100,000	0.3
**Case Fatality rate**	Level 1>10%	1.0
	Level 2: 1-10%	0.8
	Level 3: <1%	0.4
**Vaccine Efficacy**	Level 1: >80%	1.0

Criteria	Levels	Score
	Level 2: 60-79%	0.8
	Level 3: <60%	0.55
**Population at risk**	Level 1: >10 million	1.0
	Level 2: 1 – 10 million	0.8
	Level 3: 100,000 -1 million	0.5
	Level 4: < 100,000	0.3
**Target group of Vaccination**	Level A: Children (<5 years)	1.0
	Level C: High risk group	0.8
	Level B: Women	0.7
	Level D: Adult	0.5

For peer review only - http://bmjopen.bmj.com/site/about/guidelines.xhtml

Table 5: Rank order of vaccine using only quantitative criteria (from WS-A and WS-B)

	**Incidence rate**				**CFR**			**Vaccine efficacy**			**Size of population at risk**				**Target of vaccination**				**TOTAL**	
Weight of Criteria	0.26				0.22			0.21			0.19				0.13					
Levels	L1	L2	L3	L4	L1	L2	L3	L1	L2	L3	L1	L2	L3	L4	L-A	L-B	L-C	L-D	**Sum**	**Rank**
Score of Levels	1.0	0.8	0.5	0.3	1.0	0.8	0.4	1.0	0.8	0.55	1.0	0.8	0.5	0.3	1.0	0.8	0.7	0.5		
Cholera	(0.26x1.0) 0.26				(0.22x0.8) 0.17			(0.21x0.55) 0.11			(0.19x1.0) 0.19				(0.13x1.0) 0.13				0.86	1
Typhoid	(0.26x0.8) 0.20				(0.22x0.4) 0.09			(0.21x1.0) 0.21			(0.19x1.0) 0.19				(0.13x1.0) 0.13				0.82	2
Influenza	(0.26x1.0) 0.26				(0.22x0.4) 0.09			(0.21x0.8) 0.16			(0.19x1.0) 0.19				(0.13x0.7) 0.09				0.79	3
Rotavirus	(0.26x1.0) 0.26				(0.22x0.4) 0.09			(0.21x0.55) 0.11			(0.19x1.0) 0.19				(0.13x1.0) 0.13				0.78	4
Dengue	(0.26x1.0) 0.26				(0.22x0.4) 0.09			(0.21x0.8) 0.16			(0.19x0.8) 0.15				(0.13x0.7) 0.09				0.75	5
Japanese encephalitis	(0.26x0.3) 0.08				(0.22x1.0) 0.22			(0.21x1.0) 0.21			(0.19x0.8) 0.15				(0.13x0.7) 0.09				0.74	6
HPV	(0.26x0.5) 0.13				(0.22x0.4) 0.09			(0.21x1.0) 0.21			(0.19x0.8) 0.15				(0.13x0.8) 0.10				0.68	7

*Data from performance matrix (Table 2) were combined with the scores for different levels (Table 4) to estimated the scores for each vaccine. These were then multiplied with weights (Table 3) to calculate overall scores, which were then used for ranking

For peer review only

For peer review only - http://bmjopen.bmj.com/site/about/guidelines.xhtml

Table 6: Ranking of vaccine on experts judgement in WS-C

Vaccine	Mean ranking from WS-C	Ranking from the analysis of WS-A and WS-B	Final ranking after deliberation in WS-C*
Japanese Encephalitis	2	6	1
HPV	1	7	2
Rotavirus	3	4	3
Cholera	5	1	4
Typhoid	4	2	5
Dengue	7	5	6
Influenza	6	3	7

*including information on cost-effectiveness and outbreak potential

For peer review only

BMJ Open

Priority Setting of Vaccine Introduction in Bangladesh: A Multi-Criteria Decision Analysis Study

Journal:	*BMJ Open*
Manuscript ID	bmjopen-2021-054219.R1
Article Type:	Original research
Date Submitted by the Author:	12-Nov-2021
Complete List of Authors:	Haider, Mohammad; Mahidol University, Health Technology Assessment Program; Government of Bangladesh Ministry of Health and Family Welfare, Directorate General of Health Services<br>Youngkong, Sitaporn; Mahidol University, Health Technology Assessment Program; Mahidol University, Social and Administrative Pharmacy Division, Department of Pharmacy, Faculty of Pharmacy<br>Thavorncharoensap, Montarat; Mahidol University, Health Technology Assessment Program; Mahidol University, Social and Administrative Pharmacy Division, Department of Pharmacy, Faculty of Pharmacy<br>Thokala, Praveen; The University of Sheffield, Health Economics and Decision Science; School of Health and Related Research (ScHARR)
**Primary Subject Heading**:	Health policy
Secondary Subject Heading:	Evidence based practice, Public health
Keywords:	HEALTH ECONOMICS, Health policy < HEALTH SERVICES ADMINISTRATION & MANAGEMENT, INFECTIOUS DISEASES, PUBLIC HEALTH

SCHOLARONE™
Manuscripts

I, the Submitting Author has the right to grant and does grant on behalf of all authors of the Work (as defined in the below author licence), an exclusive licence and/or a non-exclusive licence for contributions from authors who are: i) UK Crown employees; ii) where BMJ has agreed a CC-BY licence shall apply, and/or iii) in accordance with the terms applicable for US Federal Government officers or employees acting as part of their official duties; on a worldwide, perpetual, irrevocable, royalty-free basis to BMJ Publishing Group Ltd ("BMJ") its licensees and where the relevant Journal is co-owned by BMJ to the co-owners of the Journal, to publish the Work in this journal and any other BMJ products and to exploit all rights, as set out in our licence.

The Submitting Author accepts and understands that any supply made under these terms is made by BMJ to the Submitting Author unless you are acting as an employee on behalf of your employer or a postgraduate student of an affiliated institution which is paying any applicable article publishing charge ("APC") for Open Access articles. Where the Submitting Author wishes to make the Work available on an Open Access basis (and intends to pay the relevant APC), the terms of reuse of such Open Access shall be governed by a Creative Commons licence – details of these licences and which Creative Commons licence will apply to this Work are set out in our licence referred to above.

Other than as permitted in any relevant BMJ Author's Self Archiving Policies, I confirm this Work has not been accepted for publication elsewhere, is not being considered for publication elsewhere and does not duplicate material already published. I confirm all authors consent to publication of this Work and authorise the granting of this licence.

For peer review only

Manuscript Title: Priority Setting of Vaccine Introduction in Bangladesh: A Multi-Criteria Decision Analysis Study

Sabbir Haider,

Mahidol University Health Technology Assessment (MUHTA) Graduate Program, Bangkok, Thailand
Directorate General of Health Services, Ministry of Health and Family Welfare, Dhaka, Bangladesh

Sitaporn Youngkong, PhD,

Mahidol University Health Technology Assessment (MUHTA) Graduate Program, Bangkok, Thailand
Social and Administrative Pharmacy Division, Department of Pharmacy, Faculty of Pharmacy, Mahidol University, Bangkok, Thailand

Montarat Thavorncharoensap, PhD,

Mahidol University Health Technology Assessment (MUHTA) Graduate Program, Bangkok, Thailand
Social and Administrative Pharmacy Division, Department of Pharmacy, Faculty of Pharmacy, Mahidol University,
Bangkok, Thailand

Praveen Thokala, PhD,

Health Economics and Decision Science,
School of Health and Related Research (ScHARR),
University of Sheffield,
Sheffield, England, UK

Contact information for corresponding author:

Sitaporn Youngkong,
Social and Administrative Pharmacy Division, Department of Pharmacy, Faculty of Pharmacy, Mahidol University,
447 SriAyudthaya Rd, Rachathewi, Bangkok 10400, Thailand
e-mail: sitaporn.you@mahidol.edu

Contributors: SH conceived and design the study, collected data, analyze the results and drafted the manuscript. SY and PT helped in the study design, data analysis, interpretation of results and reviewed the manuscript. MT contributed in design the study and reviewed the

For peer review only

manuscript. All authors discussed the findings of the study, edited and approved the manuscript.

Financial disclosure: This research work was supported by funding from Mahidol University and the International Decision Support Initiative (iDSI) through the doctoral study in Mahidol University Health Technology Assessment (MUHTA) Graduate Program. This work was produced as part of the iDSI (www.idsihealth.org), which supports countries to get the best value for money from health spending. iDSI [grant number-OPP1087363]: receives funding support from the Bill & Melinda Gates Foundation and the UK Department for International Development.

Disclaimer: The findings, interpretations and conclusions expressed in this article do not necessarily reflect the views of the funding agencies

Ethical consideration: Ethical clearance of this study was obtained from the Bangladesh Medical and Research Council (BMRC), Bangladesh (BMRC/NREC/206-2019/108 Date: 27-06-2019).

Competing interest statement: The authors declared no potential conflicts of interest with respect to the research and authorship.

Data sharing: Data is available by emailing corresponding author Sitaporn Youngkong, email-sitaporn.you@mahidol.edu

Word count: Abstract: 212 ; Manuscript: 3249

Number Figures: 0

Number of Tables: 6

Appendix/Supplemental materials: 0

For peer review only

Manuscript Title: Priority Setting of Vaccine Introduction in Bangladesh: A Multi Criteria Decision Analysis Study

ABSTRACT

Objective

To prioritize vaccines for introduction in Bangladesh.

Methods

Multi-criteria decision analysis (MCDA) process was applied to prioritize the potential vaccines for introduction in Bangladesh. A set of criteria was identified, weighted, and assigned scores by relevant stakeholders (n=14) during workshop A. The performance matrix of the data of vaccines against the criteria set was constructed and validated with the experts in workshop B (n=6). The vaccines were ranked and then appraised by another group of stakeholders (n=10) in workshop C, and the final workshop D involved dissemination of the findings to decision makers (n=28).

Results

Five criteria including incidence rate, case fatality rate, vaccine efficacy, size of population at risk and type of population at risk were used quantitatively to evaluate and to score the vaccines. Two other criteria, cost-effectiveness and outbreak potentiality, were considered qualitatively. Upon deliberation, Japanese Encephalitis (JE) vaccine was ranked top to be introduced in Bangladesh.

Conclusions

This study presents the first application of MCDA to support the vaccine prioritization in Bangladesh health system, based on systematic evidence-based decision-making. The national policy makers agreed to introduce JE vaccine in the national vaccine benefit package. The policy makers approved the process of vaccine introduction in Bangladesh, and agree to use MCDA to prioritize health interventions in the country.

Key Words: multi-criteria decision analysis, MCDA, priority setting, vaccine

For peer review only

For peer review only - http://bmjopen.bmj.com/site/about/guidelines.xhtml

Strengths and limitations of the study

- Multi-criteria decision analysis has been introduced for explicit vaccine introduction decision making in Bangladesh, contributing to transparency and evidence-informed priority setting process.
- Participation of wide range of stakeholders in this MCDA study ensured the transparency and accountability of decision-making, which is essential for a fair priority setting process.
- Data on the vaccines on the different criteria were gathered from systematic evidence synthesis and validated with experts, and good practice MCDA guidance was followed to elicit the preferences and rank the list of vaccines for introduction in the Bangladesh government benefit package.
- Different sets of stakeholders took part in the three workshops, resulting in a lack of consistent group of stakeholders (and hence values/preferences) throughout the MCDA process.
- Stakeholders from private sectors and representatives of patient groups were not involved in the process, leading to uncertainty in accountability of the results to those stakeholders.

For peer review only - http://bmjopen.bmj.com/site/about/guidelines.xhtml

INTRODUCTION

Vaccination is the most effective public health measure to prevent infectious diseases.[1 2] Governments in developing countries prefer to invest in vaccination programs which can be financially sustainable.[3-5] While countries often consider cost-effectiveness, this should not be the only criterion for the selection of any intervention.[6 7] Different criteria, such as disease severity, effectiveness, accessibility, quality of care and equity, should play significant role in priority setting in healthcare.[8]

Decision on new vaccines to be included in the benefit package is complex.[9] There are systematic and evidence-based methods,[10] using priority setting to allocate the scarce resources to meet increasing demands.[11] Multi-Criteria Decision Analysis (MCDA) is one such approach which evaluates different options considering multiple criteria in explicit manner,[12] to aid decision makers to take fair decisions.[13] MCDA can be a useful approach to support inclusion of health interventions in the benefit pacakage.[7]

Vaccine preventable diseases such as dengue, human papillomavirus (HPV), influenza, japanese encephalitis (JE), and typhoid, are prevalent in Bangladesh.[14-18] These diseases can be prevented by the introduction of new or underused vaccines by the government of Bangladesh. However, as new vaccines have considerable budget impact, it is not clear which of those should be prioritised.[19] In the past, decision-making for vaccine introduction has been ad-hoc but there is increasing interest in prioritsation systematically evaluating multiple criteria.[19]

As such, we conducted a study applying MCDA to prioritise vaccines for introduction. This is the first study on prioritization of health interventions to make better use limited resources in Bangladesh, which provided the national decision makers of ministry of health with a scientific and evidence-based systematic process incorporating multiple criteria and involving related key stakeholders. This paper describes the methods and results of the study, along with discussion and conclusions.

METHODS

We followed the steps outlined in good practice guidelines for the use of MCDA in health care.[20 21] As stakeholder involvement is key, we conducted four workshops (between October 2019 and January 2020) with the relevant stakeholders during the MCDA process. Ethical clearance of this study was obtained from the Bangladesh Medical and Research Council (BMRC) and informed written consent was obtained from the stakeholders

For peer review only - http://bmjopen.bmj.com/site/about/guidelines.xhtml

participating in the workshops. The steps and the workshops are described in further detail below.

1. Identifying the list of potential vaccines for introduction

The potential vaccines for prioritization were identified from the recommendations of World Health Organization (WHO), Gavi the vaccine alliance, and centers for disease control and prevention in the USA (CDC-US). Vaccines which were currently in the expanded program on immunization (EPI) program of the neighbouring countries were also considered. From these sources, vaccines which were not yet introduced in Bangladesh were identified as potential vaccines to be evaluated.

2. Selecting criteria for vaccine introduction in Bangladesh

A three step-process was used to select criteria for vaccine introduction in Bangladesh. First, a systematic review was conducted to identify all potential criteria for vaccine introduction in Bangladesh, which is described elsewhere in detail.[22] Second, from this long list of criteria, core team of three public health experts of Bangladesh (including the lead author, SH) excluded criteria that cannot be quantified (e.g. political will) and those that were mentioned less frequently.

Finally, the potential criteria list were ranked in a workshop A (WS-A) in October 2019, to identify the key criteria to be used for prioritisation off vaccines. Stakeholders (n=14) included paediatricians (n=1), public health experts (n=3), virologists (n=2), epidemiologists (n=4), health economics (n=1) and health system experts (n=3). In terms of affiliation, these stakeholders (n=14) were from directorate offices (n=4), technical institutes (n=4), non-government ofrganizations (NGOs) (n=3), national immunization technical advisory group (NITAG) (n=2), and health professional associations (n=1). The criteria, along with their definitions, were presented to the stakeholders who were then asked to rank each criteria from '1 to 10', where '1' is the most preferable and '10' is the least preferable criterion. The ranked order of criteria was transformed into ranking weight using rank order centroid (ROC) method.[23] Criteria were ranked based on the mean ROC weight, and the stakeholders selected a set of criteria by consensus to be used in prioritising vaccines.

For peer review only - http://bmjopen.bmj.com/site/about/guidelines.xhtml

3. Weighting and scoring

In the same workshop (WS-A), the stakeholders weighted the criteria using direct rating methods. Stakeholders discussed and then agreed by consensus to assign points to each criterion in a scale of 0-100, where '0' depicted the least important, and '100' represented the most important. To calculate the weights, the points assigned for each criterion was normalized (i.e. by dividing the points allocated to each criterion by the sum of points of all criteria) using Equation 1.[24 25]

$$w_i = p_i / \sum p_i \qquad \text{Equation 1}$$

where, w_i = normalized weight of criterion i

i = index of criterion

p_i = points allocated to each criterion

For scoring, the levels of criteria were identified by the core team from literature review and expert opinion. These were presented to the stakeholders in WS-A, who then assigned scores to the levels in each criteria individually. The stakeholders deliberated on these individual scores and assigned scores to each level of the criteria by consensus. The range of scores were between 0 to 1, where, '0' depicted the lowest score, and '1' represented the highest score.

4. Gathering evidence

Data for the potential vaccines were collected from literature reviews, databases and reports from key organisations such as EPI, Communicable Disease Control of Directorate General of Health Services (CDC-DGHS), Institute of Epidemiology, Disease Control and Research (IEDCR), and International Centre for Diarrhoeal Disease and Research, Bangladesh (icddr,b). A performance matrix was constructed, which presents data of each vaccine against the set of criteria. Then, workshop B (WS-B) was arranged in November 2019, to validate the data with a group of public health and vaccine experts in the country (n=6), i.e., public health experts who were working in the disease surveillance (n=2), DGHS (n=2), health economics unit (HEU) (n=1) and NITAG (n=1). After reviewing and validation, they signed off on the performance matrix.

For peer review only - http://bmjopen.bmj.com/site/about/guidelines.xhtml

5. Rank ordering the potential vaccines

The scores for the different levels from the WS-A were combined with the validated performance matrix from the WS-B to calculate the scores for each vaccine on the different criteria. Then, using the additive method[21] (see Equation 2),[26] the scores of each vaccine corresponding to the criteria level was multiplied by the weight of each criterion to calculate the total scores of each potential vaccine. The vaccines were ranked based on the total scores of each vaccine, with the highest total score ranked top, and the next highest total second, and so on.

$$V_j = \sum C_{ij} * W_i \qquad \text{Equation 2}$$

Where V_j is the total value for alternative *i*,
C_{ij} is the score of level on criteria *i* and
W_i is the weight attached to criteria *i*.

6. Appraising the rank of vaccines

Workshop C (WS-C) to appraise the vaccines was conducted in December 2019 with the experts in the area of vaccination (n=10), i.e., epidemiologists (n=2), virologists (n=3), infectious disease specialists (n=2), surveillance experts (n=1), and members of the vaccination policy program (n=2). The performance matrix of potential vaccines was provided in paper-based format and the stakeholders were asked to assign the rank to the seven potential vaccines individually, where '1' is the most preferable vaccine. The mean rank of each vaccine were calculated from the ranks provided by each stakeholder, using the ROC method (equation 1).

The ranking analysis of vaccines retrieved from step 5 based on findings from WS-A and WS-B were then presented to the stakeholders, along with the evidence of the cost-effectiveness and outbreak potentiality of each vaccine. Stakeholders then considered all this information and deliberated to reach consensus on a final ranking of vaccines.

7. Application of vaccine prioritization process in Bangladesh health system

A final workshop D (WS-D) was organised in January 2020 with the policy makers (n=28) working in vaccine decision-making, vaccination program implementation, vaccine related research, and disease surveillance. The stakeholders were representatives from the

For peer review only

For peer review only - http://bmjopen.bmj.com/site/about/guidelines.xhtml

ministry of health (n=12), directorate office of health (n=9), development partners (n=2), health professional associations (n=2), and NGOs (n=3). This workshop involved dissemination of the whole vaccine prioritization process (including the selection process of criteria, identification of vaccines and the MCDA methods), along with the findings. The list of ranked ordered vaccines was submitted to the ministry of health of Bangladesh for further policy action.

Patient and public involvement

In this study, patients were not involved or participated.

RESULTS

1. The list of potential vaccines for introduction in Bangladesh

WHO recommended 23 vaccines for introduction as routine vaccination globally, whereas the CDC-US recommended 16 vaccines and Gavi the vaccine alliance provided support against 16 infectious diseases.[27-29] Bangladesh so far introduced 10 vaccines in their benefit package and two additional vaccines for the Haj pilgrimage travellers. Therefore, there were 11 vaccines not included yet in the Bangladesh health benefit package. After discussion among the core team and vaccine experts, vaccines were excluded for four conditions: tick-borne encephalitis, and yellow fever as Bangladesh lacked incidence data for these diseases, and varicella and hepatitis-A virus vaccines as they were not included in the benefit package of the neighbouring countries. Seven vaccines (i.e., cholera, dengue, typhoid, HPV, influenza, JE, and rotavirus) were selected for consideration in the priority setting process**.**

2. Prioritization criteria for vaccine introduction in Bangladesh

Sixty-seven criteria were identified in the systematic review, from which the core team identified 10 criteria as being potentially most relevant (Table 1). Definitions of these 10 criteria were derived from the literature review.[30-32]

In the workshop WS-A, stakeholders discussed the importance of each of these 10 criteria and justification for its inclusion in the set of prioritization criteria to be used for vaccine introduction in Bangladesh. Participants ranked individually first and after deliberation, consensus was achieved. Table 1 presents the mean of individual ranking using ROC method

For peer review only - http://bmjopen.bmj.com/site/about/guidelines.xhtml

and the final consensus ranking. Based on these rankings, stakeholders selected the top five criteria for vaccine prioritisation in Bangladesh (i.e., incidence rate, case fatality rate, vaccine efficacy, size of population at risk, and type of population at risk). In addition to these five quantitative criteria, stakeholders also decided to discuss qualitatively 'outbreak potentiality' and 'cost-effectiveness' criteria. These two criteria were not weighted or scored explicitly, but the vaccines performance against these criteria were used in deliberative discussions.

3. Performance matrix

The data on the performance of each of seven vaccines against the prioritization criteria were presented in Table 2. The table presents the data on the five quantitative criteria used for weighting and scoring, as well as the two qualitative criteria that were used in deliberative discussions.

4. Weighting and scoring

The participants of the WS-A consensually assigned 100 points to the criterion of incidence rate and four other criteria were assigned points in accordance, with the least important criterion, 'type of population at risk' assigned 50 points. The weight of each criterion was calculated by using the linear normalization method, where weights of 'incidence rate' and 'size of population at risk' were 0.26 and 0.19, respectively as presented in Table 3.

In the same workshop WS-A, the stakeholders assigned scores for the levels of each of the five criteria by consensus, using direct rating methods. The scores for the different levels of each criterion are presented in Table 4.

5. Rank ordering the potential vaccines

After combining the findings from Tables 2-4 (i.e. the weights and scores for the different levels from WS-A, and the performance matrix validated in WS-C), the core team performed analysis of seven vaccines and produced the ranking results, where cholera vaccine was top-ranked with the highest total score of 0.34 as shown in Table 5.

For peer review only - http://bmjopen.bmj.com/site/about/guidelines.xhtml

6. Appraising the rank of vaccines

In the WS-C, the stakeholders reviewed the performance matrix and each stakeholder ranked the vaccines individually first. The mean of their individual rankings are presented in Table 6.

The results of ranking by the core team (Table 5 using findings from WS-A and WS-B) were presented along with the information on potentiality of outbreak of the diseases and cost-effectiveness (see Table 2). After considering all this information, the stakeholders adjusted the ranking by consensus and the final ranking is presented in Table 6.

7. Application of vaccine prioritization process in Bangladesh health system

Decision-makers outlined importance on the appraising new interventions scientifically and agreed to apply MCDA in the priority setting process in vaccine introduction decision making, and assigned NITAG for further prioritising vaccines for introduction in the country. They agreed on the importance of introduction on the JE vaccine as the top ranked vaccine in the government benefit package. The key personnel of ministry of health and family welfare, Bangladesh, stated –*"It is better for Bangladesh at present to have this system to prioritize vaccines in the country. Bangladesh, a low-middle income country is graduating Gavi funding. So, we have to change our decision-making process from donor influenced decision-making to self-decision-making."* They also highlighted that after selection of vaccines, country should prepare for vaccine logistics such as cold-chain capacity and other programmatic issues.

DISCUSSION

Statement of the principal findings

This study represents the first time an explicit priority setting process based on MCDA to select the vaccines to be introduced in Bangladesh. Vaccines selected for prioritisation were those which were recommended by the international organizations but not included in health benefit package of Bangladesh. Long list of multiple criteria were identified systematically from published literature, which were then shortlisted in two phases to select five quantitative criteria and two qualitative criteria for the evaluation of the vaccines. Weighting and scoring of the quantitative criteria were explicit and participatory, and the tool used for eliciting scores and weights were user friendly and well understood by the

For peer review only

For peer review only - http://bmjopen.bmj.com/site/about/guidelines.xhtml

stakeholders. The final ranking of the vaccines was determined after considering the performance matrix, which considered both quantitative criteria and qualitative criteria. The findings of the study was presented to the decision makers who agreed on the findings and the importance of using MCDA for prioritisation.

Strengths of the study, and relation to findings from other studies

Stakeholder involvement

The MCDA process was supported by different stakeholders who are involved in the decision-making process of the country. Members of the different decision-making committees (NITAG), implementing bodies (EPI and others), and health professional associations were involved in every step of the decision-making in this research. Stakeholders of implementing agencies – EPI and CDC-DGHS also participated in the deliberative process and ranking. NITAG members and members of NCIP also participated in the final decision-making workshop at ministry level. Participation of stakeholders in this research ensured the transparency and accountability of decision-making, which is essential for a fair priority setting approach.[33] This is the same as some countries, e.g., South Korea,[34] Oman, [35] Indonesia, [36] and the Netherlands [37] that ensure this transparency by involving different stakeholders during their national decision-making of vaccine introduction.

Criteria used in priority setting

Incidence rate of the disease and case fatality rate criteria weighted highly, indicating that disease burden was considered important for vaccine selection by the stakeholders. This finding is similar to other studies which suggest disease burden as the most common and important criterion considered by other low- and middle-income countries (LMICs) during national decision-making.[19 38-42] Efficacy of the vaccines was the next most important criterion suggesting that clinical effectiveness is also important.

Deliberative MCDA

The final ranking in this study was based on deliberation using the performance matrix, where the weights and scores were not explicit. Deliberation among stakeholders followed by simple ranking appears a feasible strategy for the prioritisation of vaccines for introduction in Bangladesh. Kenya and Iran choose vaccine by voting, whereas Oman, India and Netherlands choose vaccine by expert evaluation which were evidence-based but not systematic. [35 37 43 44] Korea and Thailand selected vaccine systematically and evidence-based

For peer review only - http://bmjopen.bmj.com/site/about/guidelines.xhtml

by using DELPHI and using MCDA. [34 45] Our prioritisation technique is in line with the recent consensus on the use of MCDA for HTA,[46] which recommends deliberative MCDA approach over quantitative MCDA.

Implications for policymakers

Whilst decision making around vaccines in LMICs has been driven by donor funding, our study shows that it is possible to perform prioritisation systematically using evidence-based MCDA approaches. The stakeholders decided unanimously to introduce JE vaccine in the government benefit package. Please note that the ranking of vaccines and the selection of JE vaccine is country specific and may not be applicable to other settings. It is noteworthy that a decision making on itself is a dynamic process, and some vaccine performance on some criteria used are likely to change overtime. Therefore, we suggest Bangladesh undertaking this priority setting process routinely even though most of the countries evaluate vaccine to be introduced one at a time.[39 40 43 47-50]

Limitations of the study

Different sets of stakeholders took part in the three workshops, resulting in a lack of consistent group of stakeholders (and hence values/preferences) throughout the MCDA process. The ranking from quantitative weighting and scoring (from WS-A and WS-B) was slightly different from the ranking by the stakeholders in the WS-C, who ranked the vaccines after a deliberative process reviewing the performance matrix. This may be due to the differences in the stakeholder membership between the different workshops and the underlying differences in their preferences.

Furthermore, the vaccine ranking in WS-C was finalised after considering the cost-effectiveness and the outbreak potentiality criteria, as well as the quantitative ranking. Also, the stakeholder preferences were implicit in the WS-C while they were explicitly elicited in the ranking using quantitative weighting and scoring (from WS-A and WS-B). This highlights the importance of ensuring consistent set of criteria and a consistent preference elicitation methodology throughout the MCDA process, along with a consistent group of stakeholders. In our study, the difference between the rankings was quite minimal however this may not always be the case for future studies. If the membership or the methodology changes between the different workshops, there is a possibility that the ranking may change quite substantially.

For peer review only

For peer review only - http://bmjopen.bmj.com/site/about/guidelines.xhtml

Despite the inclusion of wide variety of stakeholders, our study does not represent all stakeholders' perspectives. Stakeholders from private sectors and representatives of patient groups were not involved in the process leading to uncertainty in accountability of the results to those stakeholders.

Finally, in our study, the cost-effectiveness considerations and data of outbreak potentiality were included as qualitative criteria rather than quantitative criteria with explicit weighting and scoring. It is important to note that cost-effectiveness is not recommended as a criterion in the MCDA,[51 52] as such, a pragmatic approach was taken to consider this information qualitatively rather than weighting and scoring. Whilst decision making around vaccines has typically been driven by donor funding assurance, financial considerations are highlighted as being key by stakeholders. Given this, capacity building around economic evaluation and budget impact analysis of vaccines needs to be employed in LMICs such as Bangladesh to support evidence based priority setting combining MCDA with Value for Money (VfM) approaches. [52-54]

CONCLUSIONS

This study presents the first application of MCDA to support the vaccine prioritization in Bangladesh health system, and was based on systematic evidence-based decision-making. This research involved relevant stakeholders in priority setting process, and achieved the objectives for prioritizing the vaccine for introduction in Bangladesh in a transparent way. Policy makers agreed to introduce Japanese encephalitis vaccine in the benefit package of Bangladesh to reduce the disease burden. Government of Bangladesh can adopt this method for future vaccine introduction decision making process. Policy makers should promote the use of MCDA to prioritize interventions in healthcare, as the decision-making process can be improved using systematic MCDA approach.

For peer review only - http://bmjopen.bmj.com/site/about/guidelines.xhtml

TABLES

Table 1: Selecting criteria based on ranking from the workshop WS-A

Criteria	Rank	
	**Using mean of individuals**	**Consensus after deliberation**
Incidence rate of disease*	1	1
Case fatality rate*	2	2
Vaccine efficacy*	3	3
Size of population at risk*	5	4
Type of Target population/ Demographic consideration*	6	5
Outbreak potentiality	4	6
Cost-effectiveness	7	7
Severity of disease	8	8
Global Target	9	9
Equity	10	10

*criteria selected for vaccine prioritisation in Bangladesh

For peer review only - http://bmjopen.bmj.com/site/about/guidelines.xhtml

Table 2: Performance matrix with data of vaccines on the criteria (after validation in WS-B)

Vaccine preventable disease	Incidence rate	Case fatality rate	Vaccine efficacy	Population at risk	Target population	Cost effectiveness**	Outbreak Potential***
	Number of new cases per 100,000 population per year	Percentage of death among the cases in a year	Effectiveness of vaccine or reduction of diseases provided by vaccine (%)	No. of population at risk of getting infection per year (in millions)	Type of population needed to be vaccinated	Cost-effectiveness results from published literature	
**Cholera**[55-58]	1640	3.0%	53	15.175	Under 5 children	cost-effective	High
**Dengue**[59-61]	3700	0.16% *	66	2.18 *	Adult/High-risk	Very cost-effective	High
**HPV**[62-65]	10.6	0.0115%	95	1.56	Woman	Highly cost-effective	Low
**Influenza**[57 66-70]	10,200	0.088%	63	15.5	High risk	cost-effective	Low
**Japanese encephalitis**[16 57 71-74]	2.7	30.0%	96.20	10.77	High risk	very cost effective	Medium
**Rotavirus**[57 75-77]	1080	0.0055%	43	15.175	Under 5 children	Very cost effective	High*
**Typhoid**[57 78-82]	280	0.30%	81.60	15.175	Under 5 children	Cost effective	Medium

For peer review only - http://bmjopen.bmj.com/site/about/guidelines.xhtml

Vaccine preventable disease	Incidence rate	Case fatality rate	Vaccine efficacy	Population at risk	Target population	Cost effectiveness**	Outbreak Potential***

* Expert opinion; **Not included in weighting and scoring, used in deliberative discussions in workshop WS-C for final rankings. Judgements on cost-effectiveness were made from conclusions from published literature which evaluated the cost-effectivenss of these vaccines in Bangladesh or similar countries. ***Not included in weighting and scoring, used in deliberative discussions in workshop WS-C for final rankings.

Table 3: Points allocated, and the calculated weights, for the criteria (from WS-A)

Criteria	Points	Weight
Incidence rate	100	0.26
Case fatality rate	85	0.22
Vaccine efficacy	80	0.21
Size of population at risk	75	0.19
Type of population at risk	50	0.13

For peer review only - http://bmjopen.bmj.com/site/about/guidelines.xhtml

For peer review only

Table 4: Scores for the levels of criteria (from WS-A)

Criteria	Levels	Score
**Incidence rate**	Level 1: >1000/100,000	1.0
	Level 2: 100-1000/100,000	0.8
	Level 3: 10-100/100,000	0.5
	Level 4: <10/100,000	0.3
**Case Fatality rate**	Level 1>10%	1.0
	Level 2: 1-10%	0.8
	Level 3: <1%	0.4
**Vaccine Efficacy**	Level 1: >80%	1.0
	Level 2: 60-79%	0.8
	Level 3: <60%	0.55
**Population at risk**	Level 1: >10 million	1.0
	Level 2: 1 – 10 million	0.8
	Level 3: 100,000 -1 million	0.5
	Level 4: < 100,000	0.3
**Target group of Vaccination**	Level A: Children (<5 years)	1.0
	Level C: High risk group	0.8
	Level B: Women	0.7
	Level D: Adult	0.5

For peer review only - http://bmjopen.bmj.com/site/about/guidelines.xhtml

Table 5: Rank order of vaccine using only quantitative criteria (from WS-A and WS-B)

	Incidence rate				CFR			Vaccine efficacy			Size of population at risk				Target of vaccination				TOTAL	
Weight of Criteria	0.26				0.22			0.21			0.19				0.13					
Levels	L1	L2	L3	L4	L1	L2	L3	L1	L2	L3	L1	L2	L3	L4	L-A	L-B	L-C	L-D	Sum	Rank
Score of Levels	1.0	0.8	0.5	0.3	1.0	0.8	0.4	1.0	0.8	0.55	1.0	0.8	0.5	0.3	1.0	0.8	0.7	0.5		
Cholera	(0.26x1.0) 0.26				(0.22x0.8) 0.17			(0.21x0.55) 0.11			(0.19x1.0) 0.19				(0.13x1.0) 0.13				0.86	1
Typhoid	(0.26x0.8) 0.20				(0.22x0.4) 0.09			(0.21x1.0) 0.21			(0.19x1.0) 0.19				(0.13x1.0) 0.13				0.82	2
Influenza	(0.26x1.0) 0.26				(0.22x0.4) 0.09			(0.21x0.8) 0.16			(0.19x1.0) 0.19				(0.13x0.7) 0.09				0.79	3
Rotavirus	(0.26x1.0) 0.26				(0.22x0.4) 0.09			(0.21x0.55) 0.11			(0.19x1.0) 0.19				(0.13x1.0) 0.13				0.78	4
Dengue	(0.26x1.0) 0.26				(0.22x0.4) 0.09			(0.21x0.8) 0.16			(0.19x0.8) 0.15				(0.13x0.7) 0.09				0.75	5
Japanese encephalitis	(0.26x0.3) 0.08				(0.22x1.0) 0.22			(0.21x1.0) 0.21			(0.19x0.8) 0.15				(0.13x0.7) 0.09				0.74	6
HPV	(0.26x0.5) 0.13				(0.22x0.4) 0.09			(0.21x1.0) 0.21			(0.19x0.8) 0.15				(0.13x0.8) 0.10				0.68	7

*Data from performance matrix (Table 2) were combined with the scores for different levels (Table 4) to estimated the scores for each vaccine. These were then multiplied with weights (Table 3) to calculate overall scores, which were then used for ranking

For peer review only - http://bmjopen.bmj.com/site/about/guidelines.xhtml

Table 6: Ranking of vaccines

Vaccine	Ranking from WS-C	Ranking from the analysis of WS-A and WS-B	Final ranking after deliberation in WS-C*
Japanese Encephalitis	2	6	1
HPV	1	7	2
Rotavirus	3	4	3
Cholera	5	1	4
Typhoid	4	2	5
Dengue	7	5	6
Influenza	6	3	7

*including consideration of information on cost-effectiveness and outbreak potential

For peer review only

For peer review only - http://bmjopen.bmj.com/site/about/guidelines.xhtml

References

1. Timmis JK, Black S, Rappuoli R. Improving accountability in vaccine decision-making. *Expert Rev Vaccines* 2017;16(11):1057-66. doi: 10.1080/14760584.2017.1382358

2. Brenzel L, Wolfson LJ, Fox-Rushby J, et al. Vaccine-Preventable Diseases—Chapter 20. *Disease Control Priorities in Developing Countries*:389-411.

3. Ehreth J. The value of vaccination: a global perspective. *Vaccine* 2003;21(27-30):4105-17.

4. Ehreth J. The global value of vaccination. *Vaccine* 2003;21(7-8):596-600.

5. Mauskopf J, Standaert B, Connolly MP, et al. Economic Analysis of Vaccination Programs: An ISPOR Good Practices for Outcomes Research Task Force Report. *Value Health* 2018;21(10):1133-49. doi: 10.1016/j.jval.2018.08.005

6. Baltussen R, Jansen MP, Mikkelsen E, et al. Priority Setting for Universal Health Coverage: We Need Evidence-Informed Deliberative Processes, Not Just More Evidence on Cost-Effectiveness. *Int J Health Policy Manag* 2016;5(11):615-18. doi: 10.15171/ijhpm.2016.83

7. Norheim OF. Ethical priority setting for universal health coverage: challenges in deciding upon fair distribution of health services. *BMC Med* 2016;14:75. doi: 10.1186/s12916-016-0624-4

8. Youngkong S, Kapiriri L, Baltussen R. Setting priorities for health interventions in developing countries: a review of empirical studies. *Trop Med Int Health* 2009;14(8):930-9. doi: 10.1111/j.1365-3156.2009.02311.x

9. Munira SL, Fritzen SA. What influences government adoption of vaccines in developing countries? A policy process analysis. *Soc Sci Med* 2007;65(8):1751-64. doi: 10.1016/j.socscimed.2007.05.054

10. Baltussen R, Niessen L. Priority setting of health interventions: the need for multi-criteria decision analysis. *Cost effectiveness and resource allocation : C/E* 2006;4:14. doi: 10.1186/1478-7547-4-14

11. Mitton C, Donaldson C. Health care priority setting: principles, practice and challenges. *Cost effectiveness and resource allocation : C/E* 2004;2(1):3. doi: 10.1186/1478-7547-2-3

12. Thokala P, Duenas A. Multiple criteria decision analysis for health technology assessment. *Value Health* 2012;15(8):1172-81. doi: 10.1016/j.jval.2012.06.015

For peer review only - http://bmjopen.bmj.com/site/about/guidelines.xhtml

13. Marsh K, Goetghebeur M, Thokala P, et al. Multi-Criteria Decision Analysis to Support Healthcare Decisions: Springer 2017.

14. Haque F, Hossain MJ, Kundu SK, et al. Cholera Outbreaks in Urban Bangladesh In 2011. *Epidemiology (Sunnyvale)* 2013;3 doi: 10.4172/2161-1165.1000126

15. Hossain MJ, Gurley ES, Montgomery S, et al. Hospital-based surveillance for Japanese encephalitis at four sites in Bangladesh, 2003-2005. *Am J Trop Med Hyg* 2010;82(2):344-9. doi: 10.4269/ajtmh.2010.09-0125

16. Paul RC, Rahman M, Gurley ES, et al. A novel low-cost approach to estimate the incidence of Japanese encephalitis in the catchment area of three hospitals in Bangladesh. *Am J Trop Med Hyg* 2011;85(2):379-85. doi: 10.4269/ajtmh.2011.10-0706

17. Hasan AZ, Saha S, Saha SK, et al. Using pneumococcal and rotavirus surveillance in vaccine decision-making: A series of case studies in Bangladesh, Armenia and the Gambia. *Vaccine* 2018;36(32 Pt B):4939-43. doi: 10.1016/j.vaccine.2018.06.001

18. Chowdhury PDP, K..K.; Haque, C.E.; Hossain, S.; Lindsay, L. R.; Dibernardo, A.; Brooks, W. A.; Drebot, M. A. Dengue seroprevalence, seroconversion and risk factors in Dhaka, Bangladesh. *PLoS Negl Trop Dis* 2017;11(3) doi: https://doi.org/10.1371/journal.

19. Uddin J, Sarma H, Bari TI, et al. Introduction of new vaccines: decision-making process in Bangladesh. *J Health Popul Nutr* 2013;31(2):211-7.

20. Marsh K, M IJ, Thokala P, et al. Multiple Criteria Decision Analysis for Health Care Decision Making--Emerging Good Practices: Report 2 of the ISPOR MCDA Emerging Good Practices Task Force. *Value Health* 2016;19(2):125-37. doi: 10.1016/j.jval.2015.12.016

21. Thokala P, Devlin N, Marsh K, et al. Multiple Criteria Decision Analysis for Health Care Decision Making--An Introduction: Report 1 of the ISPOR MCDA Emerging Good Practices Task Force. *Value Health* 2016;19(1):1-13. doi: 10.1016/j.jval.2015.12.003

22. Haider MS. Multi-Criteria Decision Analysis for Priority Setting of Vaccine Introduction in Bangladesh [PhD Thesis]. Mahidol University, 2020.

23. Roszkowska E. Rank ordering criteria weighting methods–a comparative overview. 2013

24. Podviezko A, Podvezko V. Influence of data transformation on multicriteria evaluation result. *Procedia Engineering* 2015;122:151-57.

25. Normalization techniques for multi-criteria decision making: analytical hierarchy process case study. doctoral conference on computing, electrical and industrial systems; 2016. Springer.

For peer review only - http://bmjopen.bmj.com/site/about/guidelines.xhtml

26. Marsh K, Thokala P, Mühlbacher A, et al. Incorporating Preferences and Priorities into MCDA: Selecting an Appropriate Scoring and Weighting Technique. In: Marsh K, Goetghebeur M, Thokala P, et al., eds. Multi-Criteria Decision Analysis to Support Healthcare Decisions. Cham: Springer International Publishing 2017:47-66.

27. World Health Organization. WHO recommendations for routine immunization - summary tables 2019 [updated 26 April 2019; cited 2019 21 November 2019]. Available from: https://www.who.int/immunization/policy/immunization_tables/en/ accessed 21 November, 2019 2019.

28. Gavi The Vaccine Alliance. Gavi, The Vaccine Alliance-Vaccine Support 2019 [cited 2019 21 November 2019]. Available from: https://www.gavi.org/programmes-impact/types-support/vaccine-support2019.

29. Centers for Disease Control and Prevention. Centers for Disease Control and Prevention 2019 [Available from: https://www.cdc.gov/vaccines/ accessed 21 November 2019 2019.

30. Rashid K. Hyder's Textbook of community Medicine and public Health: RHM publishers, Dhaka, 2008.

31. World Health Organization. World Health Statistics 2012 - Indicator compendium: World Health Organization 2012.

32. World Health Organization. Global reference list of 100 core health indicators: World Health Organization, 2015.

33. Daniels N. Accountability for reasonableness. *BMJ* 2000;321(7272):1300-1.

34. Choe YJ, Han OP, Cho H, et al. Prioritization of the introduction of new vaccines to the national immunization program in the Republic of Korea. *Vaccine* 2014;32(46):6049-53. doi: 10.1016/j.vaccine.2014.09.009 [published Online First: 2014/09/23]

35. Al Awaidy S. The National Committee for Vaccines Regulation and Surveillance of Vaccine-Preventable Diseases in the Sultanate of Oman: evidence-based approach and consensus decision-making. *Vaccine* 2010;28 Suppl 1:A39-41. doi: 10.1016/j.vaccine.2010.02.031

36. Hadisoemarto PF, Reich MR, Castro MC. Introduction of pentavalent vaccine in Indonesia: A policy analysis. *Health Policy Plan* 2016;31(8):1079-88. doi: 10.1093/heapol/czw038

For peer review only - http://bmjopen.bmj.com/site/about/guidelines.xhtml

37. van der Putten IM, Paulus ATG, Hiligsmann M, et al. Evidence-informed vaccine decision making: The introduction of Human Papilloma Virus (HPV) vaccination in the Netherlands. *Health Policy* 2018 doi: 10.1016/j.healthpol.2018.09.001

38. Burchett HE, Mounier-Jack S, Griffiths UK, et al. National decision-making on adopting new vaccines: a systematic review. *Health Policy Plan* 2012;27 Suppl 2:ii62-76. doi: 10.1093/heapol/czr049

39. Hadisoemarto PF, Reich MR, Castro MC. Introduction of pentavalent vaccine in Indonesia: a policy analysis. *Health Policy Plan* 2016;31(8):1079-88. doi: 10.1093/heapol/czw038 [published Online First: 2016/04/24]

40. Kant L. NTAGI subcommittee recommendations on Haemophilus influenzae type b (Hib) vaccine introduction in India. *Indian Pediatr* 2009;46(11):945-54.

41. Makinen M, Kaddar M, Molldrem V, et al. New vaccine adoption in lower-middle-income countries. *Health Policy Plann* 2012;27(SUPPL.2):ii39-ii49. doi: 10.1093/heapol/czs036

42. Van Der Putten IM, Evers SMAA, Deogaonkar R, et al. Stakeholders' perception on including broader economic impact of vaccines in economic evaluations in low and middle income countries: A mixed methods study. *BMC Public Health* 2015;15(1) doi: 10.1186/s12889-015-1638-0

43. Dawa J, Chaves SS, Ba Nguz A, et al. Developing a seasonal influenza vaccine recommendation in Kenya: Process and challenges faced by the National Immunization Technical Advisory Group (NITAG). *Vaccine* 2019;37(3):464-72. doi: 10.1016/j.vaccine.2018.11.062

44. Muliyil JP, Bhan MK, Bhattacharya SK, et al. NTAGI subcommittee recommendations on Haemophilus influenzae type b (Hib) vaccine introduction in India. *Indian Pediatr* 2009;46(11):945-54.

45. Pooripussarakul S, Riewpaiboon A, Bishai D, et al. What criteria do decision makers in Thailand use to set priorities for vaccine introduction? *BMC Public Health* 2016;16:684. doi: 10.1186/s12889-016-3382-5

46. Baltussen R, Marsh K, Thokala P, et al. Multicriteria Decision Analysis to Support Health Technology Assessment Agencies: Benefits, Limitations, and the Way Forward. *Value Health* 2019;22(11):1283-88. doi: https://doi.org/10.1016/j.jval.2019.06.014

For peer review only - http://bmjopen.bmj.com/site/about/guidelines.xhtml

47. Dorji T, Tshomo U, Phuntsho S, et al. Introduction of a National HPV vaccination program into Bhutan. *Vaccine* 2015;33(31):3726-30. doi: 10.1016/j.vaccine.2015.05.078 [published Online First: 2015/06/10]

48. Fletcher MA, Tetelboum R, Fritzell B. Time to recommend pneumococcal vaccination for all children in Europe: experience in France. *Eur J Pediatr* 2002;161 Suppl 2:S132-4. doi: 10.1007/s00431-002-1065-y [published Online First: 2002/12/21]

49. Garpenholt Ö, Fredlund H, Timpka T. Immunization against Haemophilus influenzae type b in Sweden - A study of the introduction process. *Scandinavian Journal of Public Health* 2001;29(4):271-78. doi: 10.1177/14034948010290041201

50. Ngcobo NJ, Cameron NA. The decision making process on new vaccines introduction in South Africa. *Vaccine* 2012;30 Suppl 3:C9-13. doi: 10.1016/j.vaccine.2012.04.027 [published Online First: 2012/09/04]

51. Marsh KD, Sculpher M, Caro JJ, et al. The Use of MCDA in HTA: Great Potential, but More Effort Needed. *Value Health* 2018;21(4):394-97. doi: https://doi.org/10.1016/j.jval.2017.10.001

52. Marsh K, Thokala P, Youngkong S, et al. Incorporating MCDA into HTA: challenges and potential solutions, with a focus on lower income settings. *Cost Effectiveness and Resource Allocation* 2018;16(1):43. doi: 10.1186/s12962-018-0125-8

53. Wilson EC, Peacock SJ, Ruta D. Priority setting in practice: what is the best way to compare costs and benefits? *Health Econ* 2009;18(4):467-78. doi: 10.1002/hec.1380

54. Airoldi M, Morton A, Smith J, et al. Healthcare prioritisation at the local level: a socio-technical approach. 2011

55. Khan AI, Levin A, Chao DL, et al. The impact and cost-effectiveness of controlling cholera through the use of oral cholera vaccines in urban Bangladesh: A disease modeling and economic analysis. *PLoS Negl Trop Dis* 2018;12(10):e0006652. doi: 10.1371/journal.pntd.0006652

56. World Health Organization. Cholera 2014. *Wkly Epidemiol Rec* 2015;90(40):517-44.

57. Hossain MA, Latif AHMM, Rayhan MI, et al. Population Projection of Bangladesh: Dynamics and Trends 2011-2061. Dhaka, Bangladesh: Bangladesh Bureay of Statistics, Statistics and Information Division, Ministry of Planning 2015.

58. Eisenberg MC, Robertson SL, Tien JH. Identifiability and estimation of multiple transmission pathways in cholera and waterborne disease. *J Theor Biol* 2013;324:84-102. doi: 10.1016/j.jtbi.2012.12.021

For peer review only - http://bmjopen.bmj.com/site/about/guidelines.xhtml

59. Haider S, Chaikledkaew U, Thavorncharoensap M, et al. Cost-Effectiveness of Dengue Vaccine introduction in Dhaka City, Bangladesh. HTAsiaLink. Chiang Mai, Thailand, 2018.

60. World Health Organization. Dengue vaccine: WHO position paper–July 2016. *Wkly Epidemiol Rec* 2016;91(30):349-64.

61. World Health Organization. Dengue vaccine: WHO position paper, July 2016–recommendations. *Vaccine* 2017;35(9):1200-01.

62. Mahumud RA, Gow J, Alam K, et al. Cost-effectiveness of the introduction of two-dose bi-valent (Cervarix) and quadrivalent (Gardasil) HPV vaccination for adolescent girls in Bangladesh. *Vaccine* 2020;38(2):165-72. doi: 10.1016/j.vaccine.2019.10.037

63. Services DGoH. National Strategy for Cervical Cancer Prevention and Control Bangladesh (2017-2022): Directorate General of Health Services, 2017.

64. Jit M, Brisson M, Portnoy A, et al. Cost-effectiveness of female human papillomavirus vaccination in 179 countries: a PRIME modelling study. *The Lancet Global health* 2014;2(7):e406-14. doi: 10.1016/S2214-109X(14)70237-2

65. Riesen M, Garcia V, Low N, et al. Modeling the consequences of regional heterogeneity in human papillomavirus (HPV) vaccination uptake on transmission in Switzerland. *Vaccine* 2017;35(52):7312-21. doi: 10.1016/j.vaccine.2017.10.103

66. Ahmed M, Aleem MA, Roguski K, et al. Estimates of seasonal influenza-associated mortality in Bangladesh, 2010-2012. *Influenza Other Respir Viruses* 2018;12(1):65-71. doi: 10.1111/irv.12490

67. Brooks WA, Goswami D, Rahman M, et al. Influenza is a major contributor to childhood pneumonia in a tropical developing country. *Pediatr Infect Dis J* 2010;29(3):216-21. doi: 10.1097/INF.0b013e3181bc23fd

68. Zaman K, Roy E, Arifeen SE, et al. Effectiveness of maternal influenza immunization in mothers and infants. *The New England journal of medicine* 2008;359(15):1555-64. doi: 10.1056/NEJMoa0708630 [published Online First: 2008/09/19]

69. Orenstein EW, Orenstein LA, Diarra K, et al. Cost-effectiveness of maternal influenza immunization in Bamako, Mali: A decision analysis. *PLoS One* 2017;12(2):e0171499. doi: 10.1371/journal.pone.0171499

70. van den Driessche P. Reproduction numbers of infectious disease models. *Infect Dis Model* 2017;2(3):288-303. doi: 10.1016/j.idm.2017.06.002

For peer review only

71. Touch S, Suraratdecha C, Samnang C, et al. A cost-effectiveness analysis of Japanese encephalitis vaccine in Cambodia. *Vaccine* 2010;28(29):4593-9. doi: 10.1016/j.vaccine.2010.04.086

72. World Health Organization. Japanese encephalitis vaccines: WHO position paper. *Weekly Epidemiological Record= Relevé épidémiologique hebdomadaire* 2015;90(09):69-88.

73. Tandan JB, Ohrr H, Sohn YM, et al. Single dose of SA 14-14-2 vaccine provides long-term protection against Japanese encephalitis: a case-control study in Nepalese children 5 years after immunization. drjbtandan@yahoo.com. *Vaccine* 2007;25(27):5041-5. doi: 10.1016/j.vaccine.2007.04.052

74. Estimation of parameters and basic reproductive ratio for japanese encephalitis transmission in the philippines using a sequential monte carlo filter. 2017 IEEE Conference on Control Technology and Applications (CCTA); 2017. IEEE.

75. Sarker AR, Sultana M, Mahumud RA, et al. Cost-effectiveness analysis of introducing universal childhood rotavirus vaccination in Bangladesh. *Hum Vaccin Immunother* 2018;14(1):189-98. doi: 10.1080/21645515.2017.1356962

76. Troeger C, Khalil IA, Rao PC, et al. Rotavirus Vaccination and the Global Burden of Rotavirus Diarrhea Among Children Younger Than 5 Years. *JAMA Pediatr* 2018;172(10):958-65. doi: 10.1001/jamapediatrics.2018.1960

77. Zaman K, Dang DA, Victor JC, et al. Efficacy of pentavalent rotavirus vaccine against severe rotavirus gastroenteritis in infants in developing countries in Asia: a randomised, double-blind, placebo-controlled trial. *Lancet* 2010;376(9741):615-23. doi: 10.1016/S0140-6736(10)60755-6

78. Antillon M, Bilcke J, Paltiel AD, et al. Cost-effectiveness analysis of typhoid conjugate vaccines in five endemic low- and middle-income settings. *Vaccine* 2017;35(27):3506-14. doi: 10.1016/j.vaccine.2017.05.001

79. Marchello CS, Hong CY, Crump JA. Global typhoid fever incidence: A systematic review and meta-analysis. *Clinical Infectious Diseases* 2019;68:S105-S16. doi: 10.1093/cid/ciy1094

80. Yu AT, Amin N, Rahman MW, et al. Case-Fatality Ratio of Blood Culture-Confirmed Typhoid Fever in Dhaka, Bangladesh. *J Infect Dis* 2018;218(suppl_4):S222-S26. doi: 10.1093/infdis/jiy543

For peer review only - http://bmjopen.bmj.com/site/about/guidelines.xhtml

81. Shakya M, Colin-Jones R, Theiss-Nyland K, et al. Phase 3 Efficacy Analysis of a Typhoid Conjugate Vaccine Trial in Nepal. *The New England journal of medicine* 2019;381(23):2209-18. doi: 10.1056/NEJMoa1905047

82. Pitzer VE, Bowles CC, Baker S, et al. Predicting the impact of vaccination on the transmission dynamics of typhoid in South Asia: a mathematical modeling study. *PLoS Negl Trop Dis* 2014;8(1):e2642. doi: 10.1371/journal.pntd.0002642

For peer review only

For peer review only - http://bmjopen.bmj.com/site/about/guidelines.xhtml

BMJ Open

Priority Setting of Vaccine Introduction in Bangladesh: A Multi-Criteria Decision Analysis Study

Journal:	*BMJ Open*
Manuscript ID	bmjopen-2021-054219.R2
Article Type:	Original research
Date Submitted by the Author:	18-Jan-2022
Complete List of Authors:	Haider, Mohammad; Mahidol University, Health Technology Assessment Program; Government of Bangladesh Ministry of Health and Family Welfare, Directorate General of Health Services Youngkong, Sitaporn; Mahidol University, Health Technology Assessment Program; Mahidol University, Social and Administrative Pharmacy Division, Department of Pharmacy, Faculty of Pharmacy Thavorncharoensap, Montarat; Mahidol University, Health Technology Assessment Program; Mahidol University, Social and Administrative Pharmacy Division, Department of Pharmacy, Faculty of Pharmacy Thokala, Praveen; The University of Sheffield, Health Economics and Decision Science; School of Health and Related Research (ScHARR)
**Primary Subject Heading**:	Health policy
Secondary Subject Heading:	Evidence based practice, Public health
Keywords:	HEALTH ECONOMICS, Health policy < HEALTH SERVICES ADMINISTRATION & MANAGEMENT, INFECTIOUS DISEASES, PUBLIC HEALTH

SCHOLARONE™
Manuscripts

I, the Submitting Author has the right to grant and does grant on behalf of all authors of the Work (as defined in the below author licence), an exclusive licence and/or a non-exclusive licence for contributions from authors who are: i) UK Crown employees; ii) where BMJ has agreed a CC-BY licence shall apply, and/or iii) in accordance with the terms applicable for US Federal Government officers or employees acting as part of their official duties; on a worldwide, perpetual, irrevocable, royalty-free basis to BMJ Publishing Group Ltd ("BMJ") its licensees and where the relevant Journal is co-owned by BMJ to the co-owners of the Journal, to publish the Work in this journal and any other BMJ products and to exploit all rights, as set out in our licence.

The Submitting Author accepts and understands that any supply made under these terms is made by BMJ to the Submitting Author unless you are acting as an employee on behalf of your employer or a postgraduate student of an affiliated institution which is paying any applicable article publishing charge ("APC") for Open Access articles. Where the Submitting Author wishes to make the Work available on an Open Access basis (and intends to pay the relevant APC), the terms of reuse of such Open Access shall be governed by a Creative Commons licence – details of these licences and which Creative Commons licence will apply to this Work are set out in our licence referred to above.

Other than as permitted in any relevant BMJ Author's Self Archiving Policies, I confirm this Work has not been accepted for publication elsewhere, is not being considered for publication elsewhere and does not duplicate material already published. I confirm all authors consent to publication of this Work and authorise the granting of this licence.

For peer review only

Manuscript Title: Priority Setting of Vaccine Introduction in Bangladesh: A Multi-Criteria Decision Analysis Study

Sabbir Haider,

Mahidol University Health Technology Assessment (MUHTA) Graduate Program, Bangkok, Thailand
Directorate General of Health Services, Ministry of Health and Family Welfare, Dhaka, Bangladesh

Sitaporn Youngkong, Ph.D.,

Mahidol University Health Technology Assessment (MUHTA) Graduate Program, Bangkok, Thailand
Social and Administrative Pharmacy Division, Department of Pharmacy, Faculty of Pharmacy, Mahidol University, Bangkok, Thailand

Montarat Thavorncharoensap, Ph.D.,

Mahidol University Health Technology Assessment (MUHTA) Graduate Program, Bangkok, Thailand
Social and Administrative Pharmacy Division, Department of Pharmacy, Faculty of Pharmacy, Mahidol University,
Bangkok, Thailand

Praveen Thokala, Ph.D.,

Health Economics and Decision Science,
School of Health and Related Research (ScHARR),
University of Sheffield,
Sheffield, England, UK

Contact information for the corresponding author:

Sitaporn Youngkong,
Mahidol University Health Technology Assessment (MUHTA) Graduate Program,
Bangkok, Thailand
Social and Administrative Pharmacy Division, Department of Pharmacy, Faculty of Pharmacy, Mahidol University,
447 SriAyudthaya Rd, Rachathewi, Bangkok 10400, Thailand
e-mail: sitaporn.you@mahidol.edu

Contributors: SH conceived and designed the study, collected data, analysed the results and drafted the manuscript. SY and PT helped in the study design, data analysis, interpretation of results and reviewed the manuscript. MT contributed to the design of the study and reviewed

For peer review only

the manuscript. All authors discussed the findings of the study, edited and approved the manuscript.

Financial disclosure: This research work was supported by funding from Mahidol University and the International Decision Support Initiative (iDSI) through the doctoral study in Mahidol University Health Technology Assessment (MUHTA) Graduate Program. This work was produced as part of the iDSI (www.idsihealth.org), which supports countries to get the best value for money from health spending. iDSI [grant number-OPP1087363] receives funding support from the Bill & Melinda Gates Foundation and the UK Department for International Development.

Disclaimer: The findings, interpretations and conclusions expressed in this article do not necessarily reflect the views of the funding agencies.

Ethical consideration: Ethical clearance of this study was obtained from the Bangladesh Medical and Research Council (BMRC), Bangladesh (BMRC/NREC/206-2019/108 Date: 27-06-2019).

Competing interest statement: The authors declared no potential conflicts of interest with respect to the research and authorship.

Data sharing: Data is available by emailing corresponding author Sitaporn Youngkong, email-sitaporn.you@mahidol.edu

Word count: Abstract: 205 ; Manuscript: 3757

Number Figures: 0

Number of Tables: 6

Appendix/Supplemental materials: 1

For peer review only

Manuscript Title: Priority Setting of Vaccine Introduction in Bangladesh: A Multi-Criteria Decision Analysis Study

ABSTRACT

Objective

To prioritise vaccines for introduction in Bangladesh.

Methods

Multi-criteria decision analysis (MCDA) process was used to prioritise potential vaccines for introduction in Bangladesh. A set of criteria was identified, weighted, and assigned scores by relevant stakeholders (n=14) during workshop A. The performance matrix of the data of vaccines against the criteria set was constructed and validated with the experts (n=6) in workshop B. The vaccines were ranked and appraised by another group of stakeholders (n=10) in workshop C, and the final workshop D involved the dissemination of the findings to decision-makers (n=28).

Results

Five criteria including incidence rate, case fatality rate, vaccine efficacy, size of the population at risk and type of population at risk were used quantitatively to evaluate and to score the vaccines. Two other criteria, cost-effectiveness and outbreak potentiality were considered qualitatively. Upon deliberation, the Japanese Encephalitis (JE) vaccine was ranked top to be recommended for introduction in Bangladesh.

Conclusions

Based on the MCDA results, JE vaccine is planned to be recommended to the decision makers for introduction into the national vaccine benefit package. The policy makers support the use of systematic evidence-based decision-making processes such as MCDA for vaccine introduction in Bangladesh, and to prioritise health interventions in the country.

Key Words: multi-criteria decision analysis, MCDA, priority setting, vaccine

For peer review only - http://bmjopen.bmj.com/site/about/guidelines.xhtml

Strengths and limitations of the study

- Multi-criteria decision analysis (MCDA) process was used to support vaccine introduction decision-making in Bangladesh, contributing to transparency and evidence-informed priority setting.
- Participation of a wide range of stakeholders in this MCDA study ensured the transparency and accountability of decision-making, which is essential for a fair priority setting process.
- Data on the vaccines on the different criteria were gathered from systematic evidence synthesis and validated with experts, and good practice MCDA guidance was followed to elicit the preferences and rank the list of vaccines.
- Different sets of stakeholders took part in the workshops, resulting in a lack of a consistent group of stakeholders (and hence values or preferences) throughout the MCDA process.
- Stakeholders from private sectors and representatives of patient groups were not involved in the process, leading to uncertainty in accountability of the results to those stakeholders.

For peer review only

INTRODUCTION

Vaccination is the most effective public health measure to prevent infectious diseases.[1 2] Governments in developing countries prefer to invest in vaccination programs that can be financially sustainable[3-5] and while countries often consider cost-effectiveness, this should not be the only criterion for the selection of interventions.[6 7] Different criteria, such as disease severity, effectiveness, accessibility, quality of care and equity, should be considered during healthcare priority setting.[8]

Decision-making around the introduction of new vaccines in the healthcare benefit package is complex.[9] There are systematic and evidence-based methods,[10] using priority setting to allocate the scarce resources to meet increasing demand.[11] Multi-Criteria Decision Analysis (MCDA) is one such approach which evaluates different options considering multiple criteria in an explicit manner,[12] to aid decision makers to make rational decisions.[13] MCDA can be a useful approach to support inclusion of health interventions in the benefit package.[7]

Vaccine preventable diseases such as dengue, human papillomavirus (HPV), influenza, japanese encephalitis (JE), and typhoid, are prevalent in Bangladesh.[14-18] These diseases can be prevented by the introduction of new or underused vaccines by the government of Bangladesh. However, new vaccines have considerable budget impact and need to be prioritised for introduction into the benefit package.[19] In the past, decision-making for vaccine introduction was ad-hoc but there is increasing interest in prioritsation using systematic evaluation of multiple criteria.[19]

As such, we conducted an MCDA study to support prioritisation of vaccines for introduction in the benefit package in Bangladesh. The aims of the study are to support prioritisation of health interventions using an evidence-based systematic process incorporating multiple criteria and involving key relevant stakeholders, and to provide national decision-makers with scientific recommendations on vaccine introduction to better use the limited resources in Bangladesh.

METHODS

We followed the steps outlined in good practice guidelines for the use of MCDA in health care.[20 21] As stakeholder involvement is key, we conducted four workshops (between October 2019 and January 2020) with the relevant stakeholders during the MCDA process.

For peer review only - http://bmjopen.bmj.com/site/about/guidelines.xhtml

Ethical clearance of this study was obtained from the Bangladesh Medical and Research Council (BMRC) and informed written consent was obtained from the stakeholders participating in the workshops. The steps and the workshops are described in further detail below.

1. Identifying the list of potential vaccines for introduction

The potential vaccines for prioritisation were identified from the recommendations of the World Health Organization (WHO), Gavi the vaccine alliance, and centers for disease control and prevention in the USA (CDC-US). Vaccines which were currently in the expanded program on immunization (EPI) program of the neighbouring countries were also considered. From these sources, vaccines that were not yet introduced in Bangladesh were identified as potential vaccines to be evaluated.

2. Selecting criteria for vaccine introduction in Bangladesh

A three step-process was used to select criteria for vaccine introduction in Bangladesh. First, a systematic review was conducted to identify all potential criteria for vaccine introduction in Bangladesh, which is described elsewhere in detail.[22] Second, from this long list of criteria, a core team of three public health experts of Bangladesh (including the lead author, SH) excluded criteria that cannot be quantified (e.g. political will) and those that were mentioned less frequently.

Finally, the potential criteria list was ranked in workshop A (WS-A) in October 2019, to identify the key criteria to be used for vaccine prioritisation. Stakeholders (n=14) included paediatricians (n=1), public health experts (n=6), virologists (n=2), epidemiologists (n=4) and health economists (n=1). In terms of affiliation, these stakeholders (n=14) were from directorate office (n=4), technical institutes (n=4), non-government organizations (NGOs) (n=3), national immunization technical advisory group (NITAG) (n=2), and health professional associations (n=1). The criteria, along with their definitions, were presented to the stakeholders (Supplementary A) who were then asked to rank each criterion from '1 to 10', where '1' was the most preferable and '10' was the least preferable criterion . The ranked order of criteria was transformed into ranking weight using the rank order centroid (ROC) method.[23] Criteria were ranked based on the mean ROC weight, and the stakeholders selected a set of criteria by consensus to be used in the prioritisation of vaccines.

For peer review only - http://bmjopen.bmj.com/site/about/guidelines.xhtml

3. Weighting and scoring

In the same workshop (WS-A), the stakeholders weighted the criteria using direct rating methods. Stakeholders discussed and then agreed by consensus to assign points to each criterion on a scale of 0-100, where '0' depicted the least important, and '100' represented the most important. To calculate the weights, the points assigned for each criterion was normalized (i.e., by dividing the points allocated to each criterion by the sum of points of all criteria) using Equation 1.[24 25]

$$w_i = \ p_i / \sum p_i \qquad \text{Equation 1}$$

where, w_i = normalized weight of criterion i

i = index of criterion

p_i = points allocated to each criterion

For scoring, the levels of criteria were identified by the core team from literature review and expert opinion. These were presented to the stakeholders in WS-A, who then assigned scores to the levels in each criterion individually. The stakeholders then deliberated on these individual scores and assigned scores to each level of the criterion by consensus. The range of scores was between 0 to 1, where, '0' depicted the lowest score, and '1' represented the highest score.

4. Gathering evidence

Data for the potential vaccines were collected from databases and reports from key organisations such as EPI, Communicable Disease Control of Directorate General of Health Services (CDC-DGHS), Institute of Epidemiology, Disease Control and Research (IEDCR), and International Centre for Diarrhoeal Disease and Research, Bangladesh (icddr,b). A performance matrix was constructed, which presents data for each vaccine against the set of criteria. Then, workshop B (WS-B) was arranged in November 2019, to validate the data with a group of public health and vaccine experts in the country (n=6), i.e., public health experts who were working in the disease surveillance (n=2), DGHS (n=2), health economics unit

For peer review only

(HEU) (n=1) and NITAG (n=1). After reviewing and validation, they signed off on the performance matrix.

5. Rank ordering the potential vaccines

The scores for the different levels from the WS-A were combined with the validated performance matrix from the WS-B to calculate the scores for each vaccine on the different criteria. Then, using the additive method[21] (see Equation 2),[26] the scores of each vaccine corresponding to the criteria level were multiplied by the weight of each criterion (from WS-A) to calculate the total scores of each potential vaccine. The vaccines were ranked based on the total scores of each vaccine, with the highest total score ranked top, and the next highest total second, and so on.

$$V_j = \sum C_{ij} * W_i \qquad \text{Equation 2}$$

Where V_j is the total value for alternative j,

C_{ij} is the score of alternative j on criteria i, and

W_i is the weight attached to criteria i.

6. Appraising the rank of vaccines

Workshop C (WS-C) was conducted in December 2019 to appraise the vaccines. Stakeholders included the experts in the area of vaccination (n=10), i.e., epidemiologists (n=2), virologists (n=3), infectious disease specialists (n=2), surveillance experts (n=1), and members of the vaccination policy program (n=2). The performance matrix of potential vaccines was provided in a paper-based format (Supplementary B) and the stakeholders were asked to assign the rank to the seven potential vaccines individually, where '1' was the most preferable vaccine and "7" was the least preferable vaccine. The mean rank of each vaccine was calculated from the ranks provided by each stakeholder, using the ROC method.[23]

The ranking analysis of vaccines retrieved from step 5 (based on findings from WS-A and WS-B) were then presented to the stakeholders, along with the evidence of the cost-effectiveness and outbreak potentiality of each vaccine. Stakeholders then considered all this information and deliberated to reach a consensus on a final ranking of vaccines.

For peer review only - http://bmjopen.bmj.com/site/about/guidelines.xhtml

7. Application of vaccine prioritisation process in Bangladesh health system

A final workshop D (WS-D) was organised in January 2020 with the policy makers (n=28) working in vaccine decision-making, vaccination program implementation, vaccine related research, and disease surveillance. The stakeholders were representatives from the ministry of health (n=12), the directorate office of health (n=9), development partners (n=2), health professional associations (n=2), and NGOs (n=3). This workshop involved the dissemination of the whole vaccine prioritisation process (including the selection of criteria, identification of vaccines and the MCDA methods), along with the findings.

Patient and public involvement

Patients and the general public were not involved in this study.

RESULTS

1. The list of potential vaccines for introduction in Bangladesh

WHO recommended 23 vaccines for routine vaccination globally, while the CDC-US recommended 16 vaccines and Gavi the vaccine alliance provided support against 16 infectious diseases.[27-29] Bangladesh so far introduced 10 vaccines in their benefit package and two additional vaccines for the Haj pilgrimage travellers. Therefore, there were 11 vaccines not included yet in the Bangladesh health benefit package. After discussion among the core team and vaccine experts, four vaccines were excluded: tick-borne encephalitis and yellow fever as Bangladesh lacked incidence data for these diseases, and varicella and hepatitis-A virus vaccines as they were not included in the benefit package of the neighboring countries. Seven vaccines (i.e., cholera, dengue, typhoid, HPV, influenza, JE, and rotavirus) were then selected for consideration in the priority setting process**.**

2. Prioritisation criteria for vaccine introduction in Bangladesh

Sixty-seven criteria were identified in the systematic review, from which the core team identified 10 criteria as being potentially most relevant (Table 1). Definitions of these 10 criteria were derived from the literature review.[30-32]

For peer review only - http://bmjopen.bmj.com/site/about/guidelines.xhtml

In the workshop WS-A, stakeholders discussed the importance of each of these 10 criteria and justification for inclusion in the set of prioritisation criteria to be used for vaccine introduction in Bangladesh. Participants ranked individually first and after deliberation, consensus was achieved. Table 1 presents the mean of individual ranking using ROC method and the final consensus ranking. Based on these rankings, stakeholders selected the top five criteria for vaccine prioritisation in Bangladesh (i.e., incidence rate, case fatality rate, vaccine efficacy, size of population at risk, and type of population at risk). In addition to these five quantitative criteria, stakeholders also decided to include two qualitative criteria: 'outbreak potentiality' and 'cost-effectiveness'. These two criteria were not weighted or scored explicitly, but were used in deliberative discussions.

3. Performance matrix

The data on the performance of each of seven vaccines against the prioritisation criteria are presented in Table 2. The table presents data on the five quantitative criteria used for weighting and scoring, as well as the two qualitative criteria that were used in deliberative discussions. It should be noted that expert opinion (from WS-B) was used when there was no data available from published literature.

As shown in Table 2, influenza and dengue fever have the highest incidence among adults or high-risk groups but with relatively low case fatality rates. JE, on the other hand, has a relatively low incidence but with high case fatality rate (almost a third of patients dying from the condition). Among children, cholera and rotavirus seem to be with the highest incidence and cholera with a mortality rate of 3%. Vaccine efficacy seems to be excellent for JE and HPV (both above 90%), quite good for typhoid (above 80%), moderate for dengue and influenza (around 65%), and average for cholera (53%) and rotavirus (43%). All the vaccines seemed to be cost-effective or highly cost-effective. Finally, outbreak potential seems high for dengue, cholera and rotavirus.

4. Weighting and scoring

The participants of the WS-A consensually assigned 100 points to the criterion of 'incidence rate' and four other criteria were assigned points in accordance, with the least important criterion, 'type of population at risk' assigned 50 points. The weight of each criterion was calculated by using the normalization method, and the weight of 'incidence rate' was

For peer review only - http://bmjopen.bmj.com/site/about/guidelines.xhtml

estimated as 0.26, as presented in Table 3. 'Case fatality rate' and 'vaccine efficacy' were weighted similarly (0.22 and 0.21, respectively), 'size of the population at risk' had a weight of 0.19, and 'type of population at risk' had the lowest weight (0.13).

In the same workshop (WS-A), the stakeholders assigned scores for the different levels of the five criteria by consensus, using direct rating methods. For continuous criteria such as 'incidence rate', 'case fatality rate', 'vaccine efficacy' and 'size of the population at risk', the scores were assigned based on the levels of measures (e.g. scores of 1, 0.8 and 0.55 for three levels for vaccine efficacy based on whether efficacy is > 80%, 60-80% or <60%), while the scores for categorical criteria such 'type of population at risk' were based on the categories (e.g. scores of 1, 0.8, 0.7 and 0.5 for children, high-risk groups, women and adults, respectively). The scores for the different levels of each criterion are presented in Table 4.

5. Rank ordering the potential vaccines

After combining the findings from Tables 2-4 to estimate the score and weights (i.e., the weights from WS-A, and the scores by combining the different levels from WS-A with the data from performance matrix validated in WS-B), the core team performed analysis of seven vaccines and produced the ranking results, as shown in Table 5. Cholera vaccine was top-ranked with the highest total score of 0.86 primarily because it affects children, has a high incidence rate, high case fatality rate and with high size of population at risk. Despite having effective vaccines, JE and HPV ranked bottom (with scores of 0.74 and 0.68, respectively) because they have a low incidence rate and low size of population at risk.

6. Appraising the rank of vaccines

In the WS-C, the stakeholders reviewed the performance matrix and each stakeholder ranked the vaccines individually first. The mean of their individual rankings are presented in Table 6. Based on the deliberations of performance matrix, the stakeholders in WS-C ranked HPV, JE and rotavirus, as the first, second and third, respectively. The stakeholders discussed and highlighted the importance of the vaccine for women, which was why HPV was ranked as the first. Then, they gave priority to vaccines with high incidence rate and high case fatality rate; therefore, JE and rotavirus vaccines were ranked next highest. This contrasts with the findings from the quantitative MCDA exercise by the core team (see Table

For peer review only - http://bmjopen.bmj.com/site/about/guidelines.xhtml

5 using findings from WS-A and WS-B), which suggested cholera, typhoid and influenza as the top three ranking vaccines.

The results of ranking by the core team (Table 5) were then presented to the stakeholders in WS-C, along with the information on the potentiality of outbreak of the diseases and cost-effectiveness (see Table 2). After considering all this information, the stakeholders adjusted the ranking by consensus and the final ranking is presented in Table 6. HPV, JE and rotavirus still remained top three but the ranking order changed with JE, HPV and rotavirus being first, second and third, respectively.

7. Application of vaccine prioritisation process in Bangladesh health system

After dissemination of the findings, the policy makers agreed on the importance of appraising new interventions scientifically and supported the use of MCDA in the priority setting process for vaccine introduction decision making. The key personnel of the ministry of health and family welfare, Bangladesh, stated –*"It is better for Bangladesh at present to have this system to prioritise vaccines in the country. Bangladesh, a low-middle income country is graduating Gavi funding. So, we have to change our decision-making process from donor influenced decision-making to self-decision-making."* Based on the MCDA results, JE vaccine is planned to be recommended to the decision makers for introduction into the national vaccine benefit package. They also highlighted that after the selection of vaccines, the country should prepare for vaccine logistics such as cold-chain capacity and other programmatic issues.

DISCUSSION

Summary of the study

This study represents the first time an explicit priority setting process based on MCDA was used for the priortisation of vaccines in Bangladesh. Vaccines selected for prioritisation were those which were recommended by the international organizations but not included in health benefit package of Bangladesh. The potential multiple criteria were identified systematically from published literature, and shortlisted in two phases to select five quantitative criteria and two qualitative criteria for the evaluation of the vaccines. Weighting and scoring of the quantitative criteria were explicit and participatory, and the tools used for eliciting scores and weights were user friendly and well understood by the stakeholders. The

For peer review only

final ranking of the vaccines was determined after deliberative discussions based on the performance matrix, which considered both quantitative criteria and qualitative criteria.

Statement of the principal findings

Through this explicit MCDA approach, JE vaccine was placed as the top ranked vaccine and is planned to be recommended to the decision makers for introduction into the national vaccine benefit package. The policy makers support the use of systematic evidence-based decision-making processes such as MCDA for vaccine introduction in Bangladesh, and to prioritise health interventions in the country.

Strengths of the study, and comparison to findings from other studies

Stakeholder involvement

The MCDA process was supported by different stakeholders. Members of the different decision-making committees (NITAG), implementing bodies (EPI and others), and health professional associations were involved in every step of this study. Stakeholders of implementing agencies – EPI and CDC-DGHS also participated in the deliberative process and ranking. NITAG members and members of national committee for immunization practices (NCIP) also participated in the final decision-making workshop at the ministry level. Participation of stakeholders in this study ensured the transparency and accountability of decision-making, which is essential for a fair priority setting approach.[33] The importance of involving different stakeholders during the decision-making of vaccine introduction is also highlighted in other countries such as South Korea,[34] Oman,[35] Indonesia,[36] and the Netherlands[37].

Criteria used in priority setting

Incidence rate of the disease and case fatality rate criteria were weighted highly, indicating that disease burden was considered important for vaccine selection by the stakeholders. This finding is similar to other studies which suggest disease burden as the most common and important criterion considered by other low and middle-income countries (LMICs) during national decision-making. [19 38-42] Efficacy of the vaccines was weighted as the next most important criterion suggesting that clinical effectiveness is also important.

For peer review only - http://bmjopen.bmj.com/site/about/guidelines.xhtml

Deliberative MCDA

The final ranking in this study was based on deliberation using the performance matrix, where the weights and scores were not explicit. Despite the lack of explicit weighting and scoring, deliberative discussions are considered to be a very important part of MCDA process as it allows a shared understanding of the data, criteria and priorities. Deliberation among stakeholders followed by simple ranking appears a feasible strategy for the prioritisation of vaccines for introduction in Bangladesh and other LMICs. Kenya and Iran selected vaccines by voting, whereas Oman, India and Netherlands selected vaccines by expert evaluation which were evidence-based but not systematic. [35 37 43 44] Korea and Thailand selected vaccines systematically via evidence-based deliberation using DELPHI and MCDA techniques. [34 45] Recent consensus on the use of MCDA for HTA,[46] recommends deliberative MCDA approach over quantitative MCDA. Furthermore, a recent study by WHO encouraged weighting and scoring as they help streamline the deliberative discussions.[47] The methods used in our study, where the stakeholders deliberated the results from the quantitative MCDA and the performance matrix before finalising the ranking of vaccines, are in line with these recommendations.

Implications for policymakers

Whilst decision-making around vaccines in LMICs has been driven by donor funding, our study shows that it is possible to perform prioritisation systematically using evidence-based MCDA approaches. Based on the results of the MCDA study, the top ranked JE vaccine is planned to be recommended to the decision makers for introduction into the national vaccine benefit package. Please note that the ranking of vaccines and the selection of JE vaccine is country specific and may not be applicable to other settings. It is noteworthy that decision-making itself is a dynamic process, and vaccine performance on some criteria are likely to change over time. Therefore, we recommend Bangladesh undertake this priority setting process routinely even though most of the countries evaluate vaccines to be introduced once.[39 40 43 48-51]

Limitations of the study

Different sets of stakeholders took part in the three workshops, resulting in a lack of a consistent group of stakeholders (and hence values/preferences) throughout the MCDA process. The ranking from quantitative weighting and scoring (from WS-A and WS-B) was

For peer review only - http://bmjopen.bmj.com/site/about/guidelines.xhtml

different to the ranking by the stakeholders in the WS-C, who ranked the vaccines after a deliberative process. This may be due to the differences in the stakeholder membership between the different workshops and the underlying differences in their preferences.

Furthermore, the vaccine ranking in WS-C was finalised after considering the cost-effectiveness and the outbreak potentiality criteria, as well as the quantitative ranking. Also, the stakeholder preferences were implicit in the WS-C while they were explicitly elicited in the ranking using quantitative weighting and scoring (from WS-A and WS-B). This highlights the importance of ensuring a consistent set of criteria and a consistent preference elicitation methodology throughout the MCDA process, along with a consistent group of stakeholders. If the membership, the criteria set or the methodology changes between the different workshops, there is a possibility that the ranking may change quite substantially.

Despite the inclusion of a wide variety of stakeholders, our study does not represent all stakeholders' perspectives. Stakeholders from private sectors and representatives of patient groups were not involved in the process leading to uncertainty in accountability of the results to those stakeholders.

Finally, in our study, the cost-effectiveness considerations and data of outbreak potentiality were included as qualitative criteria rather than quantitative criteria with explicit weighting and scoring. It is important to note that cost-effectiveness is not recommended as a criterion in the MCDA,[52 53] as such, a pragmatic approach was taken to consider this information qualitatively rather than weighting and scoring. Whilst decision-making around vaccines has typically been driven by donor funding assurance, financial considerations are highlighted as being key by stakeholders. Capacity building around economic evaluation and budget impact analysis of vaccines is needed in LMICs such as Bangladesh to support evidence based priority setting combining MCDA with Value for Money (VfM) approaches. [53-55]

CONCLUSIONS

This study presents the first application of MCDA to support vaccine prioritisation in Bangladesh health system. This study involved relevant stakeholders in priority setting process and achieved the objectives of prioritising the vaccines for introduction in Bangladesh in a transparent way, using systematic evidence-based decision-making. JE vaccine was placed as the top ranked vaccine and is planned to be recommended to the decision makers for

For peer review only - http://bmjopen.bmj.com/site/about/guidelines.xhtml

introduction into the national vaccine benefit package. The use of MCDA to prioritise interventions in healthcare should be promoted as the decision-making process can be improved using systematic approaches.

For peer review only

For peer review only - http://bmjopen.bmj.com/site/about/guidelines.xhtml

TABLES

Table 1: Selecting criteria based on ranking from the workshop WS-A

Criteria	Rank	
	Using the mean of individuals	Consensus after deliberation
Incidence rate of disease*	1	1
Case fatality rate*	2	2
Vaccine efficacy*	3	3
Size of population at risk*	5	4
Type of population at risk*	6	5
Outbreak potentiality	4	6
Cost-effectiveness	7	7
Severity of disease	8	8
Global Target	9	9
Equity	10	10

*criteria selected for vaccine prioritisation in Bangladesh

For peer review only - http://bmjopen.bmj.com/site/about/guidelines.xhtml

Table 2: Performance matrix with data of vaccines on the criteria (after validation in WS-B)

Vaccine preventable disease	Incidence rate	Case fatality rate	Vaccine efficacy	Size of Population at risk	Type of population at risk	Cost effectiveness**	Outbreak Potentiality***
	Number of new cases per 100,000 population per year	Percentage of death among the cases in a year	Effectiveness of vaccine or reduction of diseases provided by vaccine (%)	No. of population at risk of getting infection per year (in millions)	Type of population needed to be vaccinated	Cost-effectiveness results from published literature	
**Cholera**[56-59]	1640	3.0%	53	15.175	Under 5 children	cost-effective	High
**Dengue**[60-62]	3700	0.16% *	66	2.18 *	Adult/High-risk	Very cost-effective	High
**HPV**[63-66]	10.6	0.0115%	95	1.56	Woman	Highly cost-effective	Low
**Influenza**[58 67-71]	10,200	0.088%	63	15.5	High risk	cost-effective	Low
**Japanese encephalitis**[16 58 72-75]	2.7	30.0%	96.2	10.77	High risk	very cost effective	Medium
**Rotavirus**[58 76-78]	1080	0.0055%	43	15.175	Under 5 children	Very cost effective	High*
**Typhoid**[58 79-83]	280	0.30%	81.60	15.175	Under 5 children	Cost effective	Medium

* Expert opinion; **Not included in weighting and scoring, used in deliberative discussions in workshop WS-C for final rankings. Judgements on cost-effectiveness were made from conclusions from published literature which evaluated the cost-effectivenss of these vaccines in Bangladesh or similar countries. ***Not included in weighting and scoring, used in deliberative discussions in workshop WS-C for final rankings.

For peer review only - http://bmjopen.bmj.com/site/about/guidelines.xhtml

Table 3: Points allocated, and the calculated weights, for the criteria (from WS-A)

Criteria	Points	Weight
Incidence rate	100	0.26
Case fatality rate	85	0.22
Vaccine efficacy	80	0.21
Size of population at risk	75	0.19
Type of population at risk	50	0.13

For peer review only - http://bmjopen.bmj.com/site/about/guidelines.xhtml

Table 4: Scores for the levels of criteria (from WS-A)

Criteria	Levels	Score
**Incidence rate**	Level 1: >1000/100,000	1.0
	Level 2: 100-1000/100,000	0.8
	Level 3: 10-100/100,000	0.5
	Level 4: <10/100,000	0.3
**Case Fatality rate**	Level 1>10%	1.0
	Level 2: 1-10%	0.8
	Level 3: <1%	0.4
**Vaccine Efficacy**	Level 1: >80%	1.0
	Level 2: 60-79%	0.8
	Level 3: <60%	0.55
**Size of Population at risk**	Level 1: >10 million	1.0
	Level 2: 1 – 10 million	0.8
	Level 3: 100,000 -1 million	0.5
	Level 4: < 100,000	0.3
**Type of Population at risk**	Level A: Children (<5 years)	1.0
	Level C: High risk group	0.8
	Level B: Women	0.7
	Level D: Adult	0.5

For peer review only - http://bmjopen.bmj.com/site/about/guidelines.xhtml

Table 5: Rank order of vaccine using only quantitative criteria (from WS-A and WS-B)

	Incidence rate				Case Fatality Rate			Vaccine efficacy			Size of population at risk				Type of population at risk				TOTAL	
Weight of Criteria	0.26				0.22			0.21			0.19				0.13					
Levels	L1	L2	L3	L4	L1	L2	L3	L1	L2	L3	L1	L2	L3	L4	L-A	L-B	L-C	L-D	**Sum**	**Rank**
Score of Levels	1.0	0.8	0.5	0.3	1.0	0.8	0.4	1.0	0.8	0.55	1.0	0.8	0.5	0.3	1.0	0.8	0.7	0.5		
Cholera	(0.26x1.0) 0.26				(0.22x0.8) 0.17			(0.21x0.55) 0.11			(0.19x1.0) 0.19				(0.13x1.0) 0.13				0.86	1
Typhoid	(0.26x0.8) 0.20				(0.22x0.4) 0.09			(0.21x1.0) 0.21			(0.19x1.0) 0.19				(0.13x1.0) 0.13				0.82	2
Influenza	(0.26x1.0) 0.26				(0.22x0.4) 0.09			(0.21x0.8) 0.16			(0.19x1.0) 0.19				(0.13x0.7) 0.09				0.79	3
Rotavirus	(0.26x1.0) 0.26				(0.22x0.4) 0.09			(0.21x0.55) 0.11			(0.19x1.0) 0.19				(0.13x1.0) 0.13				0.78	4
Dengue	(0.26x1.0) 0.26				(0.22x0.4) 0.09			(0.21x0.8) 0.16			(0.19x0.8) 0.15				(0.13x0.7) 0.09				0.75	5
Japanese encephalitis	(0.26x0.3) 0.08				(0.22x1.0) 0.22			(0.21x1.0) 0.21			(0.19x0.8) 0.15				(0.13x0.7) 0.09				0.74	6
HPV	(0.26x0.5) 0.13				(0.22x0.4) 0.09			(0.21x1.0) 0.21			(0.19x0.8) 0.15				(0.13x0.8) 0.10				0.68	7

*Data from performance matrix (Table 2) were combined with the scores for different levels (Table 4) to estimated the scores for each vaccine. These were then multiplied with weights (Table 3) to calculate overall scores, which were then used for ranking

For peer review only - http://bmjopen.bmj.com/site/about/guidelines.xhtml

Table 6: Ranking of vaccines

Vaccine	Ranking from WS-C	Ranking from the analysis of WS-A and WS-B	Final ranking after deliberation in WS-C*
Japanese Encephalitis	2	6	1
HPV	1	7	2
Rotavirus	3	4	3
Cholera	5	1	4
Typhoid	4	2	5
Dengue	7	5	6
Influenza	6	3	7

*including consideration of information on cost-effectiveness and outbreak potential

For peer review only

For peer review only - http://bmjopen.bmj.com/site/about/guidelines.xhtml

References

1. Timmis JK, Black S, Rappuoli R. Improving accountability in vaccine decision-making. *Expert Rev Vaccines* 2017;16(11):1057-66. doi: 10.1080/14760584.2017.1382358

2. Brenzel L, Wolfson LJ, Fox-Rushby J, et al. Vaccine-Preventable Diseases—Chapter 20. *Disease Control Priorities in Developing Countries*:389-411.

3. Ehreth J. The value of vaccination: a global perspective. *Vaccine* 2003;21(27-30):4105-17.

4. Ehreth J. The global value of vaccination. *Vaccine* 2003;21(7-8):596-600.

5. Mauskopf J, Standaert B, Connolly MP, et al. Economic Analysis of Vaccination Programs: An ISPOR Good Practices for Outcomes Research Task Force Report. *Value Health* 2018;21(10):1133-49. doi: 10.1016/j.jval.2018.08.005

6. Baltussen R, Jansen MP, Mikkelsen E, et al. Priority Setting for Universal Health Coverage: We Need Evidence-Informed Deliberative Processes, Not Just More Evidence on Cost-Effectiveness. *Int J Health Policy Manag* 2016;5(11):615-18. doi: 10.15171/ijhpm.2016.83

7. Norheim OF. Ethical priority setting for universal health coverage: challenges in deciding upon fair distribution of health services. *BMC Med* 2016;14:75. doi: 10.1186/s12916-016-0624-4

8. Youngkong S, Kapiriri L, Baltussen R. Setting priorities for health interventions in developing countries: a review of empirical studies. *Trop Med Int Health* 2009;14(8):930-9. doi: 10.1111/j.1365-3156.2009.02311.x

9. Munira SL, Fritzen SA. What influences government adoption of vaccines in developing countries? A policy process analysis. *Soc Sci Med* 2007;65(8):1751-64. doi: 10.1016/j.socscimed.2007.05.054

10. Baltussen R, Niessen L. Priority setting of health interventions: the need for multi-criteria decision analysis. *Cost effectiveness and resource allocation : C/E* 2006;4:14. doi: 10.1186/1478-7547-4-14

11. Mitton C, Donaldson C. Health care priority setting: principles, practice and challenges. *Cost effectiveness and resource allocation : C/E* 2004;2(1):3. doi: 10.1186/1478-7547-2-3

12. Thokala P, Duenas A. Multiple criteria decision analysis for health technology assessment. *Value Health* 2012;15(8):1172-81. doi: 10.1016/j.jval.2012.06.015

For peer review only - http://bmjopen.bmj.com/site/about/guidelines.xhtml

13. Marsh K, Goetghebeur M, Thokala P, et al. Multi-Criteria Decision Analysis to Support Healthcare Decisions: Springer 2017.

14. Haque F, Hossain MJ, Kundu SK, et al. Cholera Outbreaks in Urban Bangladesh In 2011. *Epidemiology (Sunnyvale)* 2013;3 doi: 10.4172/2161-1165.1000126

15. Hossain MJ, Gurley ES, Montgomery S, et al. Hospital-based surveillance for Japanese encephalitis at four sites in Bangladesh, 2003-2005. *Am J Trop Med Hyg* 2010;82(2):344-9. doi: 10.4269/ajtmh.2010.09-0125

16. Paul RC, Rahman M, Gurley ES, et al. A novel low-cost approach to estimate the incidence of Japanese encephalitis in the catchment area of three hospitals in Bangladesh. *Am J Trop Med Hyg* 2011;85(2):379-85. doi: 10.4269/ajtmh.2011.10-0706

17. Hasan AZ, Saha S, Saha SK, et al. Using pneumococcal and rotavirus surveillance in vaccine decision-making: A series of case studies in Bangladesh, Armenia and the Gambia. *Vaccine* 2018;36(32 Pt B):4939-43. doi: 10.1016/j.vaccine.2018.06.001

18. Chowdhury PDP, K..K.; Haque, C.E.; Hossain, S.; Lindsay, L. R.; Dibernardo, A.; Brooks, W. A.; Drebot, M. A. Dengue seroprevalence, seroconversion and risk factors in Dhaka, Bangladesh. *PLoS Negl Trop Dis* 2017;11(3) doi: https://doi.org/10.1371/journal.

19. Uddin J, Sarma H, Bari TI, et al. Introduction of new vaccines: decision-making process in Bangladesh. *J Health Popul Nutr* 2013;31(2):211-7.

20. Marsh K, M IJ, Thokala P, et al. Multiple Criteria Decision Analysis for Health Care Decision Making--Emerging Good Practices: Report 2 of the ISPOR MCDA Emerging Good Practices Task Force. *Value Health* 2016;19(2):125-37. doi: 10.1016/j.jval.2015.12.016

21. Thokala P, Devlin N, Marsh K, et al. Multiple Criteria Decision Analysis for Health Care Decision Making--An Introduction: Report 1 of the ISPOR MCDA Emerging Good Practices Task Force. *Value Health* 2016;19(1):1-13. doi: 10.1016/j.jval.2015.12.003

22. Haider MS. Multi-Criteria Decision Analysis for Priority Setting of Vaccine Introduction in Bangladesh [Ph.D. Thesis]. Mahidol University, 2020.

23. Roszkowska E. Rank ordering criteria weighting methods–a comparative overview. 2013

24. Podviezko A, Podvezko V. Influence of data transformation on multicriteria evaluation result. *Procedia Engineering* 2015;122:151-57.

For peer review only - http://bmjopen.bmj.com/site/about/guidelines.xhtml

25. Normalization techniques for multi-criteria decision making: analytical hierarchy process case study. doctoral conference on computing, electrical and industrial systems; 2016. Springer.

26. Marsh K, Thokala P, Mühlbacher A, et al. Incorporating Preferences and Priorities into MCDA: Selecting an Appropriate Scoring and Weighting Technique. In: Marsh K, Goetghebeur M, Thokala P, et al., eds. Multi-Criteria Decision Analysis to Support Healthcare Decisions. Cham: Springer International Publishing 2017:47-66.

27. World Health Organization. WHO recommendations for routine immunization - summary tables 2019 [updated 26 April 2019; cited 2019 21 November 2019]. Available from: https://www.who.int/immunization/policy/immunization_tables/en/ accessed 21 November, 2019 2019.

28. Gavi The Vaccine Alliance. Gavi, The Vaccine Alliance-Vaccine Support 2019 [cited 2019 21 November 2019]. Available from: https://www.gavi.org/programmes-impact/types-support/vaccine-support.

29. Centers for Disease Control and Prevention. Centers for Disease Control and Prevention 2019 [Available from: https://www.cdc.gov/vaccines/ accessed 21 November 2019 2019.

30. Rashid K. Hyder's Textbook of community Medicine and public Health: RHM publishers, Dhaka, 2008.

31. World Health Organization. World Health Statistics 2012 - Indicator compendium: World Health Organization 2012.

32. World Health Organization. Global reference list of 100 core health indicators: World Health Organization, 2015.

33. Daniels N. Accountability for reasonableness. *BMJ* 2000;321(7272):1300-1.

34. Choe YJ, Han OP, Cho H, et al. Prioritization of the introduction of new vaccines to the national immunization program in the Republic of Korea. *Vaccine* 2014;32(46):6049-53. doi: 10.1016/j.vaccine.2014.09.009 [published Online First: 2014/09/23]

35. Al Awaidy S. The National Committee for Vaccines Regulation and Surveillance of Vaccine-Preventable Diseases in the Sultanate of Oman: evidence-based approach and consensus decision-making. *Vaccine* 2010;28 Suppl 1:A39-41. doi: 10.1016/j.vaccine.2010.02.031

For peer review only - http://bmjopen.bmj.com/site/about/guidelines.xhtml

36. Hadisoemarto PF, Reich MR, Castro MC. Introduction of pentavalent vaccine in Indonesia: A policy analysis. *Health Policy Plan* 2016;31(8):1079-88. doi: 10.1093/heapol/czw038

37. van der Putten IM, Paulus ATG, Hiligsmann M, et al. Evidence-informed vaccine decision making: The introduction of Human Papilloma Virus (HPV) vaccination in the Netherlands. *Health Policy* 2018 doi: 10.1016/j.healthpol.2018.09.001

38. Burchett HE, Mounier-Jack S, Griffiths UK, et al. National decision-making on adopting new vaccines: a systematic review. *Health Policy Plan* 2012;27 Suppl 2:ii62-76. doi: 10.1093/heapol/czr049

39. Hadisoemarto PF, Reich MR, Castro MC. Introduction of pentavalent vaccine in Indonesia: a policy analysis. *Health Policy Plan* 2016;31(8):1079-88. doi: 10.1093/heapol/czw038 [published Online First: 2016/04/24]

40. Kant L. NTAGI subcommittee recommendations on Haemophilus influenzae type b (Hib) vaccine introduction in India. *Indian Pediatr* 2009;46(11):945-54.

41. Makinen M, Kaddar M, Molldrem V, et al. New vaccine adoption in lower-middle-income countries. *Health Policy Plann* 2012;27(SUPPL.2):ii39-ii49. doi: 10.1093/heapol/czs036

42. Van Der Putten IM, Evers SMAA, Deogaonkar R, et al. Stakeholders' perception on including broader economic impact of vaccines in economic evaluations in low and middle income countries: A mixed methods study. *BMC Public Health* 2015;15(1) doi: 10.1186/s12889-015-1638-0

43. Dawa J, Chaves SS, Ba Nguz A, et al. Developing a seasonal influenza vaccine recommendation in Kenya: Process and challenges faced by the National Immunization Technical Advisory Group (NITAG). *Vaccine* 2019;37(3):464-72. doi: 10.1016/j.vaccine.2018.11.062

44. Muliyil JP, Bhan MK, Bhattacharya SK, et al. NTAGI subcommittee recommendations on Haemophilus influenzae type b (Hib) vaccine introduction in India. *Indian Pediatr* 2009;46(11):945-54.

45. Pooripussarakul S, Riewpaiboon A, Bishai D, et al. What criteria do decision makers in Thailand use to set priorities for vaccine introduction? *BMC Public Health* 2016;16:684. doi: 10.1186/s12889-016-3382-5

For peer review only - http://bmjopen.bmj.com/site/about/guidelines.xhtml

46. Baltussen R, Marsh K, Thokala P, et al. Multicriteria Decision Analysis to Support Health Technology Assessment Agencies: Benefits, Limitations, and the Way Forward. *Value Health* 2019;22(11):1283-88. doi: https://doi.org/10.1016/j.jval.2019.06.014

47. Botwright S, Giersing BK, Meltzer MI, et al. The CAPACITI Decision-Support Tool for National Immunization Programs. *Value Health* 2021;24(8):1150-57. doi: 10.1016/j.jval.2021.04.1273

48. Dorji T, Tshomo U, Phuntsho S, et al. Introduction of a National HPV vaccination program into Bhutan. *Vaccine* 2015;33(31):3726-30. doi: 10.1016/j.vaccine.2015.05.078 [published Online First: 2015/06/10]

49. Fletcher MA, Tetelboum R, Fritzell B. Time to recommend pneumococcal vaccination for all children in Europe: experience in France. *Eur J Pediatr* 2002;161 Suppl 2:S132-4. doi: 10.1007/s00431-002-1065-y [published Online First: 2002/12/21]

50. Garpenholt Ö, Fredlund H, Timpka T. Immunization against Haemophilus influenzae type b in Sweden - A study of the introduction process. *Scandinavian Journal of Public Health* 2001;29(4):271-78. doi: 10.1177/14034948010290041201

51. Ngcobo NJ, Cameron NA. The decision making process on new vaccines introduction in South Africa. *Vaccine* 2012;30 Suppl 3:C9-13. doi: 10.1016/j.vaccine.2012.04.027 [published Online First: 2012/09/04]

52. Marsh KD, Sculpher M, Caro JJ, et al. The Use of MCDA in HTA: Great Potential, but More Effort Needed. *Value Health* 2018;21(4):394-97. doi: https://doi.org/10.1016/j.jval.2017.10.001

53. Marsh K, Thokala P, Youngkong S, et al. Incorporating MCDA into HTA: challenges and potential solutions, with a focus on lower income settings. *Cost Effectiveness and Resource Allocation* 2018;16(1):43. doi: 10.1186/s12962-018-0125-8

54. Wilson EC, Peacock SJ, Ruta D. Priority setting in practice: what is the best way to compare costs and benefits? *Health Econ* 2009;18(4):467-78. doi: 10.1002/hec.1380

55. Airoldi M, Morton A, Smith J, et al. Healthcare prioritisation at the local level: a socio-technical approach. 2011

56. Khan AI, Levin A, Chao DL, et al. The impact and cost-effectiveness of controlling cholera through the use of oral cholera vaccines in urban Bangladesh: A disease modeling and economic analysis. *PLoS Negl Trop Dis* 2018;12(10):e0006652. doi: 10.1371/journal.pntd.0006652

57. World Health Organization. Cholera 2014. *Wkly Epidemiol Rec* 2015;90(40):517-44.

For peer review only

For peer review only - http://bmjopen.bmj.com/site/about/guidelines.xhtml

58. Hossain MA, Latif AHMM, Rayhan MI, et al. Population Projection of Bangladesh: Dynamics and Trends 2011-2061. Dhaka, Bangladesh: Bangladesh Bureay of Statistics, Statistics and Information Division, Ministry of Planning 2015.

59. Eisenberg MC, Robertson SL, Tien JH. Identifiability and estimation of multiple transmission pathways in cholera and waterborne disease. *J Theor Biol* 2013;324:84-102. doi: 10.1016/j.jtbi.2012.12.021

60. Haider S, Chaikledkaew U, Thavorncharoensap M, et al. Cost-Effectiveness of Dengue Vaccine introduction in Dhaka City, Bangladesh. HTAsiaLink. Chiang Mai, Thailand, 2018.

61. World Health Organization. Dengue vaccine: WHO position paper–July 2016. *Wkly Epidemiol Rec* 2016;91(30):349-64.

62. World Health Organization. Dengue vaccine: WHO position paper, July 2016–recommendations. *Vaccine* 2017;35(9):1200-01.

63. Mahumud RA, Gow J, Alam K, et al. Cost-effectiveness of the introduction of two-dose bi-valent (Cervarix) and quadrivalent (Gardasil) HPV vaccination for adolescent girls in Bangladesh. *Vaccine* 2020;38(2):165-72. doi: 10.1016/j.vaccine.2019.10.037

64. Services DGoH. National Strategy for Cervical Cancer Prevention and Control Bangladesh (2017-2022): Directorate General of Health Services, 2017.

65. Jit M, Brisson M, Portnoy A, et al. Cost-effectiveness of female human papillomavirus vaccination in 179 countries: a PRIME modelling study. *The Lancet Global health* 2014;2(7):e406-14. doi: 10.1016/S2214-109X(14)70237-2

66. Riesen M, Garcia V, Low N, et al. Modeling the consequences of regional heterogeneity in human papillomavirus (HPV) vaccination uptake on transmission in Switzerland. *Vaccine* 2017;35(52):7312-21. doi: 10.1016/j.vaccine.2017.10.103

67. Ahmed M, Aleem MA, Roguski K, et al. Estimates of seasonal influenza-associated mortality in Bangladesh, 2010-2012. *Influenza Other Respir Viruses* 2018;12(1):65-71. doi: 10.1111/irv.12490

68. Brooks WA, Goswami D, Rahman M, et al. Influenza is a major contributor to childhood pneumonia in a tropical developing country. *Pediatr Infect Dis J* 2010;29(3):216-21. doi: 10.1097/INF.0b013e3181bc23fd

69. Zaman K, Roy E, Arifeen SE, et al. Effectiveness of maternal influenza immunization in mothers and infants. *The New England journal of medicine* 2008;359(15):1555-64. doi: 10.1056/NEJMoa0708630 [published Online First: 2008/09/19]

For peer review only - http://bmjopen.bmj.com/site/about/guidelines.xhtml

70. Orenstein EW, Orenstein LA, Diarra K, et al. Cost-effectiveness of maternal influenza immunization in Bamako, Mali: A decision analysis. *PLoS One* 2017;12(2):e0171499. doi: 10.1371/journal.pone.0171499

71. van den Driessche P. Reproduction numbers of infectious disease models. *Infect Dis Model* 2017;2(3):288-303. doi: 10.1016/j.idm.2017.06.002

72. Touch S, Suraratdecha C, Samnang C, et al. A cost-effectiveness analysis of Japanese encephalitis vaccine in Cambodia. *Vaccine* 2010;28(29):4593-9. doi: 10.1016/j.vaccine.2010.04.086

73. World Health Organization. Japanese encephalitis vaccines: WHO position paper. *Weekly Epidemiological Record= Relevé épidémiologique hebdomadaire* 2015;90(09):69-88.

74. Tandan JB, Ohrr H, Sohn YM, et al. Single dose of SA 14-14-2 vaccine provides long-term protection against Japanese encephalitis: a case-control study in Nepalese children 5 years after immunization. drjbtandan@yahoo.com. *Vaccine* 2007;25(27):5041-5. doi: 10.1016/j.vaccine.2007.04.052

75. Estimation of parameters and basic reproductive ratio for japanese encephalitis transmission in the philippines using a sequential monte carlo filter. 2017 IEEE Conference on Control Technology and Applications (CCTA); 2017. IEEE.

76. Sarker AR, Sultana M, Mahumud RA, et al. Cost-effectiveness analysis of introducing universal childhood rotavirus vaccination in Bangladesh. *Hum Vaccin Immunother* 2018;14(1):189-98. doi: 10.1080/21645515.2017.1356962

77. Troeger C, Khalil IA, Rao PC, et al. Rotavirus Vaccination and the Global Burden of Rotavirus Diarrhea Among Children Younger Than 5 Years. *JAMA Pediatr* 2018;172(10):958-65. doi: 10.1001/jamapediatrics.2018.1960

78. Zaman K, Dang DA, Victor JC, et al. Efficacy of pentavalent rotavirus vaccine against severe rotavirus gastroenteritis in infants in developing countries in Asia: a randomised, double-blind, placebo-controlled trial. *Lancet* 2010;376(9741):615-23. doi: 10.1016/S0140-6736(10)60755-6

79. Antillon M, Bilcke J, Paltiel AD, et al. Cost-effectiveness analysis of typhoid conjugate vaccines in five endemic low- and middle-income settings. *Vaccine* 2017;35(27):3506-14. doi: 10.1016/j.vaccine.2017.05.001

80. Marchello CS, Hong CY, Crump JA. Global typhoid fever incidence: A systematic review and meta-analysis. *Clinical Infectious Diseases* 2019;68:S105-S16. doi: 10.1093/cid/ciy1094

For peer review only - http://bmjopen.bmj.com/site/about/guidelines.xhtml

81. Yu AT, Amin N, Rahman MW, et al. Case-Fatality Ratio of Blood Culture-Confirmed Typhoid Fever in Dhaka, Bangladesh. *J Infect Dis* 2018;218(suppl_4):S222-S26. doi: 10.1093/infdis/jiy543
82. Shakya M, Colin-Jones R, Theiss-Nyland K, et al. Phase 3 Efficacy Analysis of a Typhoid Conjugate Vaccine Trial in Nepal. *The New England journal of medicine* 2019;381(23):2209-18. doi: 10.1056/NEJMoa1905047
83. Pitzer VE, Bowles CC, Baker S, et al. Predicting the impact of vaccination on the transmission dynamics of typhoid in South Asia: a mathematical modeling study. *PLoS Negl Trop Dis* 2014;8(1):e2642. doi: 10.1371/journal.pntd.0002642

For peer review only

For peer review only - http://bmjopen.bmj.com/site/about/guidelines.xhtml

SUPPLEMENTARY

Supplementary A: Data collection instrument of Workshop A - Ranking of criteria

Name of the participants: ____

Designation: ____

Organization: ____

Note: Pls. make rank of the following criteria - 1 to 10 (Where is the most important and 10 is the less important)

CRITERIA	DEFINITION	RANK
**Criteria of Disease**		
Case fatality rate	Percentage of death among the cases	
Incidence rate of disease	Number of new cases per 100,000 population per year	
Outbreak potentiality	Potentiality of the disease to be epidemic Potentiality will be measured by the reproduction of the disease	
Severity of disease	Symptoms of the disease; how severe the disease are in the most of the cases	
Size of population at risk	Size of the population at risk or the target population for vaccination	
Type of Target population/ Demographic consideration	Demographic consideration or Target population for the vaccination against the disease (e.g. children or female or adult)	
**Criteria of Vaccine**		
Cost-effectiveness	Cost-effectiveness of vaccine; Incremental cost-effectiveness ration (ICER) will be $/QALY gained or $/DALY avoided if the vaccine introduced in comparison to the No vaccination	
Vaccine efficacy	Effectiveness of vaccine or the percentage reduction of diseases provided by vaccine	
**Other Criteria**		
Equity	Disease occur more in economically poor people or disadvantaged population	
Global Target	Global agenda of eradication/ elimination/ control target	

For peer review only

Supplementary B: Data collection instrument of Workshop C - Ranking of vaccines

Name of the participants: ____
Designation: ____
Organization: ____

Please rank vaccines from 1-7, where 1 is most favourable and 7 is least favourable

	Criteria	Rotavirus	HPV	Cholera	JE	Typhoid	Influenza	Dengue
DISEASE	**1. Incidence rate**<br>Number of new cases per 100,000 population per year	1080/100,000	24.3/100,000	210/100,000	2.7/100,000	280/100,000	10,000/ 100,000 person year (2008)<br>6600/100,000 person year (2009)<br>17000/100,000 person year (2010)	1340-5780/ 100,000 person-season
	**2. Case fatality rate**<br>Percentage of death among the cases	0.03%<br>12.42/100,000 among < 5 years of age (Rotavirus gastroenteritis mortality) (1.24%)	1.8%<br>50%	1.5%	10-30%<br>(25%)	0.3%<br>(1%)	0.08%	2.5%
VACCINE	Type of vaccine	RV5; Live attenuated	Human Papillomaviruses Nanovalent	Shanchol	SA14-14-2 JE Vaccine	Typhoid Conjugate vaccine	Influenza trivalent vaccine: Single dose	Dengvaxia live attenuated, recombinant tetravalent vaccine
	Dosage	3 dosages	2 dosages	2 dosages	Single dose	Single dose	Single dose	3 dosages
	**3. Vaccine efficacy**<br>Effectiveness of vaccine or the percentage reduction of diseases provided by vaccine	55%<br>(40-85%)	90-100%	50-60%	95%<br>>85%	50-72%	40%<br>40-60%	66%
POPULATION	**4. Type of Target population**<br>Demographic consideration or Target population for the vaccination against the disease (e.g. children or female or adult)	Under 5 population	Girls of 10 years of age or class 5 student<br>1.54 million	1-5years of population<br>Urban and high risk population	1-15 years of children;<br>Routine immunization 9-12 months	Under 5 population	High risk group	Dhaka City population
	**5. Size of population at risk (million)**<br>Number of population need to be vaccinated or size of the population at risk or the target population for vaccination	15.17	9.17<br>1.54	13.3	7.4<br>(1-15 years of children)	15.17	15.47	2.18
	RANK							

For peer review only
